# Congenital Titinopathy: Comprehensive characterization and pathogenic insights

**DOI:** 10.1002/ana.25241

**Published:** 2018-07-27

**Authors:** Emily C. Oates, Kristi J. Jones, Sandra Donkervoort, Amanda Charlton, Susan Brammah, John E. Smith, James S. Ware, Kyle S. Yau, Lindsay C. Swanson, Nicola Whiffin, Anthony J. Peduto, Adam Bournazos, Leigh B. Waddell, Michelle A. Farrar, Hugo A. Sampaio, Hooi Ling Teoh, Phillipa J. Lamont, David Mowat, Robin B. Fitzsimons, Alastair J. Corbett, Monique M. Ryan, Gina L. O'Grady, Sarah A. Sandaradura, Roula Ghaoui, Himanshu Joshi, Jamie L. Marshall, Melinda A. Nolan, Simranpreet Kaur, Jaya Punetha, Ana Töpf, Elizabeth Harris, Madhura Bakshi, Casie A. Genetti, Minttu Marttila, Ulla Werlauff, Nathalie Streichenberger, Alan Pestronk, Ingrid Mazanti, Jason R. Pinner, Carole Vuillerot, Carla Grosmann, Ana Camacho, Payam Mohassel, Meganne E. Leach, A. Reghan Foley, Diana Bharucha‐Goebel, James Collins, Anne M. Connolly, Heather R. Gilbreath, Susan T. Iannaccone, Diana Castro, Beryl B. Cummings, Richard I. Webster, Leïla Lazaro, John Vissing, Sandra Coppens, Nicolas Deconinck, Ho‐Ming Luk, Neil H. Thomas, Nicola C. Foulds, Marjorie A. Illingworth, Sian Ellard, Catriona A. McLean, Rahul Phadke, Gianina Ravenscroft, Nanna Witting, Peter Hackman, Isabelle Richard, Sandra T. Cooper, Erik‐Jan Kamsteeg, Eric P. Hoffman, Kate Bushby, Volker Straub, Bjarne Udd, Ana Ferreiro, Kathryn N. North, Nigel F. Clarke, Monkol Lek, Alan H. Beggs, Carsten G. Bönnemann, Daniel G. MacArthur, Henk Granzier, Mark R. Davis, Nigel G. Laing

**Affiliations:** ^1^ Dubowitz Neuromuscular Centre, UCL Great Ormond Street Institute of Child Health London United Kingdom; ^2^ Institute for Neuroscience and Muscle Research Kid's Research Institute, Children's Hospital at Westmead Sydney New South Wales Australia; ^3^ Discipline of Child and Adolescent Health, Faculty of Medicine and Health The University of Sydney Sydney New South Wales Australia; ^4^ School of Biotechnology and Biomolecular Sciences Faculty of Science, The University of New South Wales Sydney New South Wales Australia; ^5^ Neuromuscular and Neurogenetic Disorders of Childhood Section, National Institute of Neurological Disorders and Stroke, National Institutes of Health Bethesda MD; ^6^ Department of Histopathology Children's Hospital at Westmead Sydney New South Wales Australia; ^7^ Electron Microscope Unit, Department of Anatomical Pathology Concord Repatriation General Hospital Concord, Sydney New South Wales Australia; ^8^ Department of Cellular and Molecular Medicine University of Arizona Tucson AZ; ^9^ National Heart and Lung Institute and MRC London Institute of Medical Science, Imperial College London London United Kingdom; ^10^ Royal Brompton and Harefield Hospitals NHS Trust London United Kingdom; ^11^ Institute for Medical Research and Centre for Medical Research University of Western Australia Nedlands Western Australia Australia; ^12^ Manton Center for Orphan Disease Research, Division of Genetics and Genomics Boston Children's Hospital, Harvard Medical School Boston MA; ^13^ Department of Radiology Westmead Hospital Sydney New South Wales Australia; ^14^ University of Sydney Western Clinical School Sydney New South Wales Australia; ^15^ Department of Neurology Sydney Children's Hospital Sydney New South Wales Australia; ^16^ School of Women's and Children's Health University of New South Wales Sydney Sydney New South Wales Australia; ^17^ Neurogenetic Unit, Department of Neurology Royal Perth Hospital Perth Western Australia Australia; ^18^ Department of Medical Genetics Sydney Children's Hospital Sydney New South Wales Australia; ^19^ Sydney Medical School The University of Sydney Sydney New South Wales Australia; ^20^ Department of Neurology Concord Repatriation General Hospital Sydney New South Wales Australia; ^21^ Department of Neurology Royal Children's Hospital Parkville Victoria Australia; ^22^ Murdoch Children's Research Institute, Royal Children's Hospital Parkville Victoria Australia; ^23^ University of Melbourne Parkville Victoria Australia; ^24^ Paediatric Neuroservices, Starship Child Health Auckland New Zealand; ^25^ Analytic and Translational Genetics Unit, Massachusetts General Hospital Boston MA; ^26^ Medical and Population Genetics, Broad Institute of Massachusetts Institute of Technology and Harvard Cambridge MA; ^27^ Research Center for Genetic Medicine, Children's National Medical Center Washington DC; ^28^ Department of Integrative Systems Biology George Washington University School of Medicine and Health Sciences Washington DC; ^29^ John Walton Muscular Dystrophy Research Centre, Institute of Genetic Medicine Newcastle University Newcastle upon Tyne United Kingdom; ^30^ Department of Clinical Genetics Liverpool Hospital Sydney New South Wales Australia; ^31^ Danish National Rehabilitation Center for Neuromuscular Diseases Aarhus Denmark; ^32^ Neuropathology Department, Hospices Civils Lyon Claude Bernard University Lyon1 France; ^33^ NeuroMyogene Institute CNRS UMR 5310, INSERM U1217 Lyon France; ^34^ Department of Neurology Washington University School of Medicine Saint Louis MO; ^35^ Department of Pathology and Immunology Washington University School of Medicine Saint Louis MO; ^36^ Cellular Pathology University Hospital Southampton NHS Foundation Trust Southampton United Kingdom; ^37^ Department of Medical Genomics Royal Prince Alfred Hospital Camperdown, Sydney New South Wales Australia; ^38^ Woman‐Mother‐Child Hospital, Hospices Civils Lyon Bron France; ^39^ Claude Bernard University Lyon1 France; ^40^ University of California, San Diego/Rady Children's Hospital San Diego CA; ^41^ Child Neurology Unit, Department of Neurology, October 12 University Hospital, Faculty of Medicine Complutense University Madrid Spain; ^42^ Division of Neurology Children's National Health System Washington DC; ^43^ Mercy Clinic Pediatric Neurology Springfield MO; ^44^ Neuromuscular Division, Departments of Neurology and Pediatrics Washington University School of Medicine Saint Louis MO; ^45^ Department of Advanced Practice Children's Medical Center of Dallas Dallas TX; ^46^ Department of Pediatrics University of Texas Southwestern Medical Center Dallas TX; ^47^ Department of Neurology and Neurotherapeutics University of Texas Southwestern Medical Center Dallas TX; ^48^ Broad Institute of Harvard and Massachusetts Institute of Technology Cambridge MA; ^49^ Program in Biological and Biomedical Sciences, Harvard Medical School Boston MA; ^50^ T. Y. Nelson Department of Neurology and Neurosurgery Children's Hospital at Westmead Sydney New South Wales Australia; ^51^ Pediatric Service, Basque Coast Hospital Center Bayonne France; ^52^ Neuromuscular Clinic and Research Unit, Department of Neurology, Rigshospitalet University of Copenhagen Copenhagen Denmark; ^53^ Department of Pediatric Neurology, Neuromuscular Reference Center, Erasmus Hospital Free University of Brussels Brussels Belgium; ^54^ Department of Pediatric Neurology, Neuromuscular Reference Center, Queen Fabiola Children's University Hospital Free University of Brussels Brussels Belgium; ^55^ Clinical Genetic Service, Department of Health Hong Kong China; ^56^ Department of Paediatric Neurology University Hospital Southampton NHS Foundation Trust Southampton United Kingdom; ^57^ Wessex Clinical Genetics Service University Hospital Southampton NHS Foundation Trust Southampton United Kingdom; ^58^ University of Exeter Medical School Exeter United Kingdom; ^59^ Department of Molecular Genetics Royal Devon and Exeter NHS Foundation Trust Exeter United Kingdom; ^60^ Department of Anatomical Pathology Alfred Hospital Melbourne Victoria Australia; ^61^ Faculty of Medicine, Nursing, and Health Sciences Monash University Melbourne Victoria Australia; ^62^ National Hospital for Neurology and Neurosurgery, UCL Institute of Neurology London United Kingdom; ^63^ Harry Perkins Institute University of Western Australia Nedlands Western Australia Australia; ^64^ Copenhagen Neuromuscular Unit and Department of Neurology, Rigshospitalet Copenhagen University Copenhagen Denmark; ^65^ Folkhälsan Institute of Genetics, Medicum University of Helsinki Helsinki Finland; ^66^ Généthon, INSERM U951, INTEGRARE Research Unit Évry France; ^67^ Department of Human Genetics Radboud University Medical Center Nijmegen the Netherlands; ^68^ Neuromuscular Research Center Tampere University and University Hospital, Neurology Tampere Finland; ^69^ Department of Medical Genetics University of Helsinki Helsinki Finland; ^70^ Vaasa Central Hospital, Department of Neurology Vaasa Finland; ^71^ Pathophysiology of Striated Muscles Laboratory, Unit of Functional and Adaptative Biology, BFA Paris Diderot University/CNRS, Sorbonne Paris Cité Paris France; ^72^ Public Hospital Network of Paris, Paris‐East Reference Center Neuromuscular Diseases, Pitié‐Salpêtrière Hospital Group Paris France; ^73^ Department of Diagnostic Genomics PathWest Laboratory Medicine WA Nedlands Western Australia Australia

## Abstract

**Objective:**

Comprehensive clinical characterization of congenital titinopathy to facilitate diagnosis and management of this important emerging disorder.

**Methods:**

Using massively parallel sequencing we identified 30 patients from 27 families with 2 pathogenic nonsense, frameshift and/or splice site *TTN* mutations *in trans*. We then undertook a detailed analysis of the clinical, histopathological and imaging features of these patients.

**Results:**

All patients had prenatal or early onset hypotonia and/or congenital contractures. None had ophthalmoplegia. Scoliosis and respiratory insufficiency typically developed early and progressed rapidly, whereas limb weakness was often slowly progressive, and usually did not prevent independent walking. Cardiac involvement was present in 46% of patients. Relatives of 2 patients had dilated cardiomyopathy. Creatine kinase levels were normal to moderately elevated. Increased fiber size variation, internalized nuclei and cores were common histopathological abnormalities. Cap‐like regions, whorled or ring fibers, and mitochondrial accumulations were also observed. Muscle magnetic resonance imaging showed gluteal, hamstring and calf muscle involvement. Western blot analysis showed a near‐normal sized titin protein in all samples. The presence of 2 mutations predicted to impact both N2BA and N2B cardiac isoforms appeared to be associated with greatest risk of cardiac involvement. One‐third of patients had 1 mutation predicted to impact exons present in fetal skeletal muscle, but not included within the mature skeletal muscle isoform transcript. This strongly suggests developmental isoforms are involved in the pathogenesis of this congenital/early onset disorder.

**Interpretation:**

This detailed clinical reference dataset will greatly facilitate diagnostic confirmation and management of patients, and has provided important insights into disease pathogenesis. Ann Neurol 2018;83:1105–1124

T*TN* (Online Mendelian Inheritance in Man database [OMIM] #188840) includes the most exons (364) and has the longest coding sequence (>100kb) of any human gene. It encodes titin, the largest protein in nature (maximum size = 4,200kDa).^1^ In striated (cardiac and skeletal) muscle, each titin molecule pairs with a second antiparallel titin molecule to span the full length of the sarcomere like two “springs in series,”^2^ forming a continuous elastic myofilament. These myofilaments provide a scaffold for sarcomere assembly during muscle development,^3^, ^4^, ^5^ generate passive tension, maintain sarcomeric structural integrity, and serve as key mechanosensing and signaling hubs (reviewed in Gautel and Djinovic‐Carugo^6^).

Titin comprises an amino‐terminal Z‐disc region, middle I‐ and A‐band regions, and a carboxy‐terminal M‐band region (Fig 1). Differential splicing of titin (particularly PEVK exons) yields alternative isoforms with different lengths and elastic properties. In humans, there is one characterized mature soleus‐derived skeletal muscle isoform, N2A.^1^, ^7^ There are also multiple mature cardiac isoforms (http://cardiodb.org/titin/titin_transcripts.php).^8^ Developmental skeletal and cardiac muscle isoforms have also been described at the protein and individual exon expression level.^9^, ^10^

**Figure 1:**
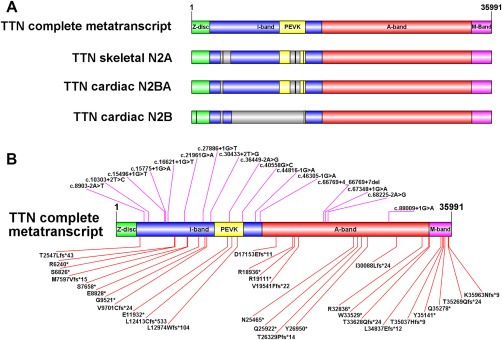
Schematic representation of titin isoforms and location of patient mutations. (A) Size (relative to number of amino acids) of the 4 main titin regions encoded by the inferred complete metatranscript (Refseq transcript NM_001267550.1), the single characterized skeletal muscle isoform N2A (Refseq transcript NM_133378.4), the principal cardiac long isoform N2BA (NM_001256850.1) and the principle cardiac short isoform N2B (NM_003319.4). The Z‐disc region of titin (green) interacts with α‐actinin, telethonin, and other Z‐disc–related proteins. The I‐band region (blue) contains multiple tandem immunoglobulin‐like domains and the “PEVK” domain (yellow), which is rich in proline (P), glutamic acid (E), valine (V), and lysine (K). The PEVK domain unravels when stretched, giving titin its elastic properties. The A‐band region (red) contains multiple myosin and C‐protein binding sites, and alternating fibronectin type III and immunoglobulin repeats that form a shape that complements myosin. The M‐band region (purple) is encoded by the last 6 exons [M‐band exon (Mex) exons 1–6 (exons 359–364)] and contains a kinase domain, immunoglobulin domains, and binding sequences for calpain 3, obscurin, MURF‐1, and numerous other proteins, along with additional unique sequences. The gray regions shown within N2A, N2BA, and N2B are those included in the metatranscript but not present within the relevant isoform. Thick black lines within the gray regions represent smaller subregions that are retained by the isoform, but are not large enough to show up as a colored segment. (B) Location of each of the mutations identified in our clinical analysis cohort (Families 1–27) mapped to the inferred complete metatranscript. Splice site mutations are shown above the transcript image. Frameshift and nonsense mutations are shown below the transcript. Supplementary Table 1 shows which mutations are included in N2A, N2BA and/or N2B. (Schematic images were created using Illustrator for Biological Sequences.)

Autosomal dominant *TTN* mutations cause 2 adult onset skeletal muscle disorders: (1) tibial muscular dystrophy (TMD; OMIM #600334)^11^ and (2) hereditary myopathy with early respiratory failure (HMERF; OMIM #603689).^12^, ^13^ All reported TMD mutations are within the final 6 (M‐band) exons. HMERF is caused by missense mutations within exon 344. Heterozygous truncating *TTN* mutations are the most common genetic cause of dilated cardiomyopathy (DCM; OMIM #604145).^14^, ^15^, ^16^

A specific Finnish founder mutation that causes TMD in heterozygous individuals causes limb girdle muscular dystrophy in homozygous individuals (OMIM #608807).^11^, ^17^ Various homozygous and compound heterozygous *TTN* mutations have also been reported in patients with childhood, adolescent, and adult onset recessive distal titinopathy,^18^, ^19^ and childhood‐juvenile onset Emery‐Dreifuss–like titinopathy.^20^

To date, 16 patients from 12 families with a recessive prenatal or infant onset form of titinopathy have been reported. Previous cases were described as early onset myopathy with fatal cardiomyopathy,^21^ centronuclear myopathy,^22^ core myopathy with heart disease,^23^ and arthrogryposis multiplex congenita with myopathy.^24^ We suggest the term “congenital titinopathy” be adopted for this disorder.^25^ Previously reported patients had a range of different causative mutations, including more difficult‐to‐interpret missense changes. Clinical features included neck, axial, and limb weakness, joint contractures, spinal and chest wall deformities, early onset respiratory insufficiency, mild facial weakness and ptosis. Congenital cardiac anomalies and/or childhood or adolescent onset DCM were common (10/16 patients). Creatine kinase (CK) levels were normal to moderately elevated. Muscle biopsies showed increased internalized and central nuclei, minicores and/or dystrophic lesions.

To facilitate diagnostic assessments and to better understand the natural history of congenital titinopathy, we analyzed the clinical, muscle pathology and imaging findings in a large international cohort with recessively inherited nonsense, frameshift and/or splice site *TTN* mutations; that is, the most pathogenically‐convincing subset of mutations. Patients with difficult‐to‐interpret missense variants were excluded, to gain the clearest possible clinical picture of this disorder.

## Subjects and Methods

### Ethical Approval and Consent

The project was approved by the Human Research Ethics Committee of the Sydney Children's Hospitals Network and by other researchers' relevant institutional review boards. Informed consent for research participation and use of clinical photographs, including 3 non‐obscured facial photographs, was obtained from patients/parents/legal guardians.

### Recruitment

Patients were ascertained from neurology clinics. The study included published and unpublished clinical data from Families 13 to 16, reported previously as 314‐1, 979‐1, 1044‐1, and 1093‐1 respectively.^22^ The Family 10 proband has also been described.^26^

### Mutation Detection, Confirmation, and Annotation

The type of massively parallel sequencing (MPS) technology used to identify each causative mutation (panel, whole exome sequencing, whole genome sequencing) is shown in Supplementary Table 1. Bioinformatics analysis pipelines focused on rare compound heterozygous or homozygous variants within known neuromuscular disease genes that were likely to be pathogenic. Variants in genes associated with cardiac abnormalities in the absence of neuromuscular pathology were not systematically analyzed in most cases.

Each mutation was Sanger‐confirmed and reported according to Human Genome Variation Society recommendations (http://varnomen.hgvs.org/) using the inferred complete *TTN* metatranscript as reference (NM_001267550.1; LRG391_t1). Exons were numbered 1 to 364 according to the LRG schema. Family members were Sanger sequenced to confirm segregation and carrier status.

The Leiden Muscular Dystrophy (http://www.dmd.nl/), ClinVar (https://www.ncbi.nlm.nih.gov/clinvar/), and Cardiodb mutation databases (http://cardiodb.org/titin/) were interrogated to identify previously reported mutations. The Exome Aggregation Consortium (ExAC) database (http://exac.broadinstitute.org/) was used to determine the frequency of each mutation in the general population. Alamut® Visual (Interactive Biosoftware, North Seattle, WA) was used to predict the impact of splice site mutations.

### Clinical Features Analysis

The clinical analysis cohort included patients with 2 Sanger‐confirmed nonsense, frameshift and/or splice site mutations shown to be *in trans* by segregation studies. The single exception was the Family 7 proband, who had a homozygous mutation and history of parental consanguinity; however, parental DNA was unavailable. The data from clinical analysis cohort members (henceforth referred to as patients) were collected from primary medical records and managing clinicians, tabulated, and the percentage of patients with each feature calculated. The denominator for each calculation (shown in Table 1 and Supplementary Table 2) was the number of patients for whom data were available for that feature, rather than total cases. As patients “unknown” for any feature are more likely to have been negative than positive for that feature, this approach may have resulted in a small overestimation of prevalence.

**Table 1:** Summary of common and clinically significant features More detailed information regarding overall findings is provided in Supplementary Table 2.

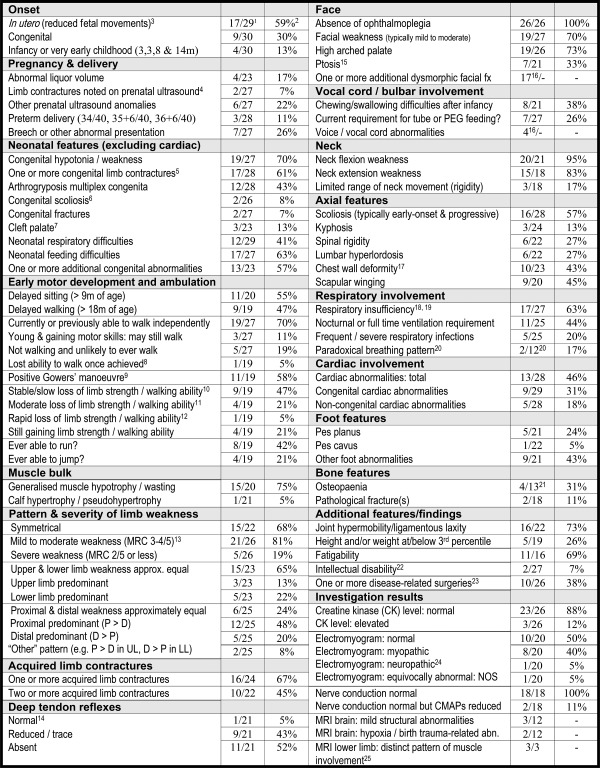

^1^Denominator is number of cohort members with data provided for that feature/item.

^2^Fraction converted to percentage of cohort members with each feature.

^3^Reduced movements from first onset of movements in pregnancy in at least 1 case; no data were available regarding fetal movements for 1 patient.

^4^Talipes noted as early as 15 weeks gestation in 1 case.

^5^A significant subset of congenital limb contractures were not noted on prenatal ultrasound.

^6^Both congenital scoliosis cases were brothers from the same family (Family 1).

^7^Submucous cleft in 2 of 3 cases; cleft in context of Pierre Robin sequence in 3rd case.

^8^Only one cohort member lost the ability to walk independently once he had achieved the ability to walk (lost at age 7 years) as a result of progressive contractures and foot deformities (“walk independently” is defined as able to walk without any assistive devices, for example, calipers).

^9^At least 1 cohort member had a positive Gowers' maneuver in early childhood but had lost this by age 7 years.

^10^“Slow” is defined as still ambulant or likely to be still ambulant after 20 years of age.

^11^“Moderate” is defined as loss or likely loss of ambulation between 10 and 20 years of age.

^12^“Rapid” is defined as loss or likely loss of ambulation before 10 years of age.

^13^Degree of muscle weakness is rated according to weakest muscle group for each cohort member. For example, if at least 1 muscle group is rated as “severe,” overall rating is “severe.”

^14^Deep tendon reflexes were normal in a single cohort member with congenital contractures (camptodactyly and talipes) but no history of limb weakness (Family 19 proband).

^15^Ptosis was typically mild, occasionally unilateral, and congenital in at least 1 cohort member.

^16^Data regarding these features were not specifically requested during the formal data collection process but were reported spontaneously in descriptive data for >1 cohort member and were therefore noteworthy. As dataset is incomplete, a denominator and percentage are not provided.

^17^In a small subset of additional cases, rotational deformity of the spine had resulted in prominence of one side of the chest wall. These were counted as “no/absent” for chest wall deformity.

^18^“Respiratory insufficiency” is defined as reduced force vital capacity and/or oxygen requirement and/or ventilation requirement.

^19^Only 2 cohort members with respiratory insufficiency had no scoliosis or chest wall deformity. One was a 9‐month‐old infant on full‐time ventilation who may yet develop truncal deformities. The other was a 32‐year‐old male who was lost to follow‐up from age 17 years.

^20^Diaphragmatic/paradoxical breathing pattern data were available for only 12 cohort members (incomplete data ascertainment). This feature could not be assessed in severely affected infants requiring full‐time ventilatory support.

^21^Many cohort members had not been screened for osteopenia at the time of ascertainment.

^22^Mild intellectual disability was reported in 2 brothers from Family 26; 1 also had attention‐deficit/hyperactivity disorder.

^23^One cohort member had undergone 4 separate surgical procedures, and several had undergone ≥2 procedures.

^24^Upper limb electromyography was felt to be neuropathic in 1 subject who died at 3 months of age. The lower limb findings in this infant were normal.

^25^Additional descriptive data regarding pattern of muscle involvement in lower limb MRI are provided in the text. CK = creatine kinase; CMAP = compound motor action potential; D = distal; fx = features; inv = involvement; LL = lower limbs; MRC = Medical Research Council scale for muscle strength; MRI = magnetic resonance imaging; NOS = not otherwise specified; P = proximal; PEG = percutaneous gastrostomy tube; UL = upper limbs.

Four additional segregation‐inconclusive families (Families 28–31) were studied, but not included in the clinical analysis cohort.

### Cardiac Isoform Analysis

The Cardiodb database (http://cardiodb.org/titin) was interrogated to determine which mutations were predicted to alter the 2 most abundant adult cardiac isoforms, N2BA and N2B. Families were stratified according to the cardiac status of affected members (“Yes” = cardiac involvement in at least 1 member), and whether each had (1) 2 mutations predicted to alter both N2BA and N2B or (2) other combinations of mutations. Significance was determined using Fisher's Exact Test.

### Western Blot Analysis

Western blot analysis of 5 patient muscle samples (Families 4, 5, 13, 15 and 16) and 2 segregation‐inconclusive cases (Families 28 and 29) was undertaken. Biopsy protein extracts were electrophoresed on 0.8% agarose gels and transferred to polyvinylidene difluoride membranes using a semidry transfer system (Bio‐Rad Laboratories, Hercules, CA). Affinity‐purified rabbit polyclonal antibodies specific for the titin N‐terminus (α‐Z1Z2; Myomedix, Neckargemünd, Germany) and C‐terminus (α‐M8M9; Myomedix) were used (1:2,500 dilution). Fluorescent secondary antibodies with infrared excitation (LI‐COR Biosciences, Lincoln, NE) were used (1:20,000 dilution). Blots were scanned with the Odyssey Infrared Imaging System (LI‐COR Biosciences).

### Splicing, cDNA and RNAseq Analysis

Direct analysis of the impact of each mutation was beyond the scope of this study; however, an *in vitro* hybrid minigene splicing assay was previously used to evaluate the transcriptional consequences of 2 splice site changes.^22^

The impact of an extended splice site deletion (Family 6: exon 317: c.66769+4_66769+7del) was evaluated using cDNA analysis. cDNA was synthesized from 1 µg of total skeletal muscle RNA using the SuperScript™ III First‐Strand Synthesis System (Invitrogen, Carlsbad, CA) for reverse transcription polymerase chain reaction (PCR), according to the manufacturer's protocol (90 minute cDNA synthesis). RNA was primed with oligo(dt) or random primers. One microliter of the resulting cDNA was used in PCR reactions. The primers were: forward 5′‐AACCGCCTCTTGAAGATGGA‐3′ (NG_011618.3 exon 315) and reverse 5′‐ TGGAGCACTCAGTTGTCACT‐3′ (exon 319).

## Results

### Demographic Information

The study cohort consisted of 30 patients from 27 families. The demographic features are described in Supplementary Table 2. Four patients were deceased at ascertainment, and 1 died during the study. Cause of death was (1) early respiratory failure (2 patients: Family 6 proband on day 1 [38 weeks gestation] and Family 18 proband at 3 months of age); (2) sudden death at age 8 years in the context of mild–moderate DCM, with arrhythmia suspected (Family 7 proband); (3) pneumonia at age 13 years (Family 10 proband); and (4) bowel cancer during early 30s (Sibling 2/Family 1). Of the 25 surviving patients, age at ascertainment ranged from 9 months to 34 years.

As only 5 patients were ≥ 18 years old (see Supplementary Table 2), it was not possible to comprehensively characterize the features or natural history of this disorder during adulthood. Younger patients were well represented.

### Mutations

The 45 different mutations identified in clinical analysis cohort members (from Families 1‐7) are shown in Figure 1 and detailed in Supplementary Table 1. Thirty‐three mutations were novel. Fifteen mutations were nonsense and 14 were frameshift. Thirteen of the 16 splice site mutations were essential/canonical (altering the first or last 2 bases of an intron). Two were exonic (altering the last base of an exon). One was a small extended splice site deletion (Family 6: c.66769+4_66769+7del). All mutations were rare (frequency < 0.1% in ExAC).

Four mutations from 8 unrelated families (Families 20–27) were within the inferred complete *TTN* metatranscript (NM_001267550.1) but not within exons that encode the N2A mature skeletal muscle isoform (NM_133378.4) or the multiple mature cardiac isoforms. In support of the pathogenicity of these “metatranscript‐only” mutations, the exon 163 nonsense mutation (p.Glu11932*) was a recurrent mutation present in 5 unrelated cohort families (Families 20, 21, 23, 24, and 25) and 1 segregation‐inconclusive family (Family 31).

Two additional mutations were also recurrent mutations present in 2 unrelated cohort families (p.Val10952Leu exonic splice mutation present in Families 4 and 13, and c.15496+1G>A present in Families 1 and 14; see Supplementary Table 1).

### Family History

Five probands had 1 affected sibling. Clinical data from 3 of these siblings (from Families 1, 20, and 26) were available for inclusion in the analysis (see Supplementary Table 1). There was no history of weakness or other neuromuscular abnormalities among the first‐degree relatives of cohort patients.

Only 19 of the carrier‐confirmed parents from 11 of 27 clinical analysis cohort families and an even smaller subset of extended family members had undergone cardiac screening (shown in Supplementary Table 1). Of specific note is that two families (Families 9 and 13) had first‐ and/or second‐degree relatives with DCM. All 3 Family 13 maternal members with DCM were heterozygous carriers. The maternally inherited mutation from this family was novel. In a third family (Family 12), a sibling pregnancy had been terminated due to congenital heart disease (fetal mutation status unknown). There was also a strong family history of cardiomyopathy (both parents and a paternal uncle) in 1 segregation‐inconclusive family (Family 29).

The 77‐year‐old Family 1 father and 2 older paternal mutation‐carrier relatives from Family 13 had additional cardiac abnormalities of unknown significance (atrial fibrillation in all 3 and mitral valve changes in 2 of 3; details in Supplementary Table 1).

Five parents (from Families 3, 4, 5, 13, and 25) carried a mutation previously reported in the DCM literature (DCM‐mutation) but had normal cardiac findings (2 parents) or had not yet had cardiac screening (3 parents; additional details in Supplementary Table 1). The Family 5 maternal carrier of a previously reported congenital titinopathy‐cardiac‐mutation (p.Gln35278*)^23^ had no cardiac involvement on recent screening.

### Clinical Features

**Figure 2:**
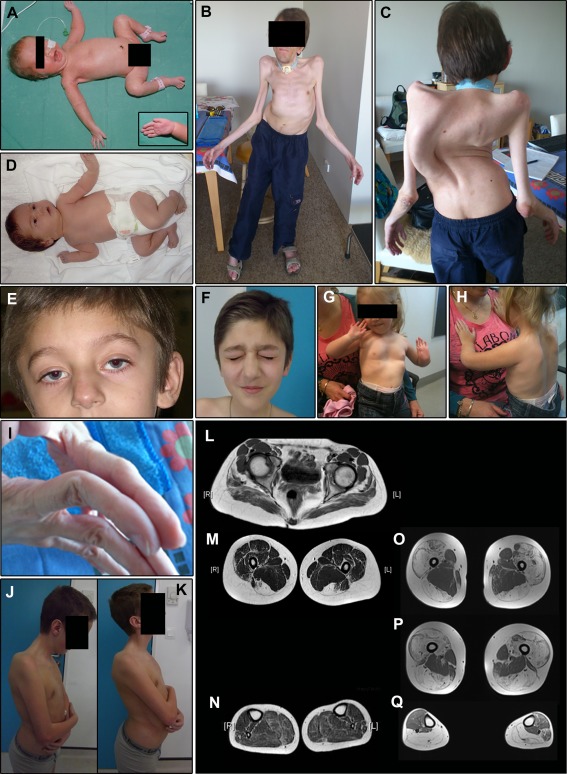
Examples of common and clinically significant features. Lower limb magnetic resonance imaging (MRI) is also shown. (A) Female Family 25 proband. The pregnancy for this infant was complicated by reduced fetal movements and prenatal ultrasound detection of limb contractures. The image shows typical “frog leg” positioning due to marked congenital hypotonia and weakness. Note also the nasogastric feeding tube and reduced palmar creases *(inset)*. This infant also had mild pulmonary stenosis and needed intubation and ventilation for a short period following delivery. (B, C) The older of the 2 affected brothers (Sibling 1) from Family 1 at age 34 years. Both brothers had congenital scoliosis, which progressed during childhood. Spinal surgery was not possible in the pictured brother due to concurrent development of severe respiratory insufficiency. His respiratory deficit was diagnosed at age 13 years following an out of hospital respiratory arrest, and he has since relied on nocturnal ventilation via a tracheostomy tube. This patient has slowly progressive mild to moderate limb weakness but remains ambulant despite significant axial involvement. He also has left ventricular cardiac dysfunction. In addition, these images demonstrate bilateral non‐congenital elbow contractures and marked generalized muscle hypotrophy. (D) Family 24 female proband who was born following a pregnancy complicated by reduced fetal movements and breech presentation. She was significantly hypotonic at birth and had congenital bilateral wrist contractures and fixed foot deformities. (E, F) Ptosis and facial weakness in the younger sibling (Sibling 2) from Family 26 during early childhood (E) and at age 12 years (F). (G, H) The same child as shown in (A) at age 2 years with a pectus excavatum chest wall deformity (G) and scapular winging (H). (I) Distal joint hypermobility in Sibling 1 from Family 1. (J, K) Reduced range of neck movement in Sibling 1 from Family 26. (L–N) Lower limb MRI result for Sibling 1 from Family 26 at age 15 years. This shows fatty infiltration of the gluteal muscles (L), severe fatty replacement of both visible hamstring muscles (complete fatty replacement of semitendinosus and incomplete but marked replacement of biceps femoris; M), relative sparing of the adductor compartment, and mild involvement of the calf and peroneal muscles (N). (O–Q) Lower limb MRI result for the Family 31 proband at age 29 years. This is one of the 4 segregation‐inconclusive cases described in this paper, but not included within the clinical analysis cohort. The features in this individual's lower limb MRI may represent the more severe end of the spectrum of muscle involvement associated with this disorder. The images show severe fatty replacement of the quadriceps and hamstrings, sparing of the adductors (also seen in the first MRI case), and severe replacement of both calves and the right peroneus, with mild fatty marbling in the anterior and lateral compartment on the left. Consent was obtained from patients/parents/legal guardians for use of the clinical photographs shown in this figure, including the non‐obscured facial photographs shown in D–F.

Common and significant clinical features, and the prevalence of each feature, are summarized in Table 1, with more detailed clinical information provided in Supplementary Table 2. Selected features are illustrated in Figure 2.

#### Pregnancy/Congenital Features

Almost 60% of patients had reduced fetal movements. Features of the disorder developed *in utero* or were present at birth in all but four cases (exceptions: symptoms/signs first noted at 3 months (two patients), 8 and 14 months). One patient (Family 19 proband) had congenital finger contractures and unilateral talipes, but no apparent weakness at birth and remains strong (current age = 21 years).

Congenital limb contractures were common (61%), and often distal (involved joint[s] are shown in Supplementary Table 2). Twelve patients (43%) had congenital contractures involving ≥ 2 areas consistent with the clinical diagnosis of arthrogryposis multiplex congenita (AMC). Congenital scoliosis and fractures were each present in 2 patients (congenital scoliosis was present in both Family 1 siblings; congenital fractures were present in Family 6 proband and Sibling 1/Family 26).

Neonatal feeding and respiratory difficulties were common (63% and 41%). Most patients required only a brief period of respiratory support after birth. Three patients (Family 12, 17, and 18 probands) had required nocturnal or all‐day ventilation from the neonatal period onward.

#### Limb Weakness, Contractures, Muscle Bulk and Motor Development

In most cases, limb weakness was predominantly proximal and symmetrical, and affected both upper and lower limbs. Severity was typically mild to moderate (Medical Research Council [MRC] 3–4/5), but sometimes severe (MRC 2/5 or less). Generalized muscle wasting was common (75%). Calf hypertrophy was rare (Family 13 proband only). Acquired limb contractures were frequent (67%), affected both proximal and distal joints, and were often multiple (see Supplementary Table 2).

A total of 19 of 30 patients could, or had previously been able to walk. Three (11%) were younger than 5 years and might still walk. Nine patients could walk fast or run, and 4 could jump. Rate of ambulatory loss was often slow (defined as “still ambulant or predicted to be still ambulant after 20 years of age”; 9/19 patients), and 4 of 19 patients were still gaining rather than losing walking ability (see Supplementary Table 2). Only 1 patient had lost the ability to walk independently (without aids) once achieved (Family 2 proband; unable to walk from age 7 years). Another initially walked with calipers but became wheelchair dependent at age 4 years (Family 5 proband). Three were experiencing increasing walking difficulties due to progressive weakness, pain, contractures, foot deformities, and/or fatigue (Family 7, 8, and 15 probands).

#### Axial Features and Respiratory Insufficiency

Neck flexion weakness was present in all but 1 of the 21 patients for whom data were available. Neck extension weakness was also common (83%). Three patients had a striking “dropped head” phenotype (Sibling1/Family 1, Sibling 1/Family 26, and Family 2 proband).^27^

Many patients (63%) had objective evidence of respiratory impairment, and 44% required nocturnal or full‐time ventilation. Scoliosis was present in 57% of patients and was typically early‐onset, rapidly progressive, and had required (or was likely to require) intervention. Chest wall deformities and scapular winging were present in 43% and 45% of patients respectively. Scoliosis, chest wall deformities, and respiratory insufficiency were often concurrent. Spinal rigidity, kyphosis, and lumbar hyperlordosis were sometimes present (6, 3, and 6 cases respectively).

#### Cardiac Abnormalities

Thirteen patients (46%) had congenital and/or early onset cardiac pathology (see Supplementary Tables 1 and 2). Nine had congenital cardiac abnormalities. Five had early onset cardiac complications including dilated cardiomyopathy (onset at 18 months and 9 years), left ventricular dysfunction (onset at 3 years and “during childhood”), or a dilated right ventricle (autopsy finding at age 13 years). Some cardiac abnormalities progressed rapidly and/or were associated with life‐threatening arrhythmias.

Two patients with structural cardiac abnormalities (Family 4 and 25 probands) had 1 reported DCM‐mutation^15^ in combination with 1 novel mutation (see Supplementary Table 1). The patient with the most severe congenital anomaly (Family 5 proband: aortic coarctation) was the only patient to have 2 previously reported cardiac mutations (a DCM‐mutation^14^ and a congenital titinopathy‐cardiac‐mutation^23^). The remaining 10 patients with cardiac involvement had no previously reported cardiac mutations.

#### Facial Features, Feeding Abnormalities and Other Notable Findings

No patient had ophthalmoplegia (based on data for 26/29 patients who survived beyond day 1 of life). High‐arched palate and mild to moderate facial weakness were common (73% and 70%). Ptosis was present in 33%, and was occasionally congenital, asymmetrical or fluctuating. A submucous cleft palate was present in 2 patients (Family 19 and 22 probands). A third patient had Pierre Robin sequence (Family 5 proband). Growth abnormalities, torticollis, facial asymmetry, and a range of dysmorphic features were noted in more than one patient (see Supplementary Table 2).

Several patients were reported to have a Noonan‐like facial appearance, sometimes in combination with other Noonan‐associated features (low posterior hairline, neck webbing, short stature); however, ascertainment of these features was incomplete.

Sucking, chewing, and/or swallowing difficulties affected 38% of patients, and 26% required supplemental tube or gastrostomy feeding.

Joint hypermobility was present in 73% of patients.

Only 2 siblings had a history of learning difficulties (mild: Family 26). Four patients had documented osteopenia, 2 with pathological fractures. None had experienced malignant hyperthermia during exposure to anesthetic agents.

### CK Levels and Electrophysiology Results

CK levels were typically normal but occasionally elevated (400–2,3241 IU/L). Nerve conduction velocities were normal. Electromyography was usually normal (50%) or myopathic (40%).

### Magnetic Resonance Imaging

Three of the 12 patients with magnetic resonance imaging (MRI) brain results had minor structural abnormalities of unknown significance (see Supplementary Table 2). Two severely affected infants had birth hypoxia or birth trauma‐related features, but normal cerebral morphology.

Descriptions of pelvic and lower limb MRI findings were available for 3 patients (at ages 3, 9, and 15 years), with original images for 1 of the 3 (Family 26, 15 years; see Fig 2L–N). All 3 cases showed paraspinal, gluteal, and global hamstring atrophy. The adductors, sartorius, and gracilis were spared or hypertrophied. In the lower limb, the gastrocnemius and soleus were variably involved. Muscle MRI signs associated with clinically similar disorders, for example *SEPN1‐* and *COL6‐*myopathies^28^, ^29^, ^30^ were absent.

Original images from a segregation‐inconclusive case at age 29 years (Family 31; see Fig 2O‐Q) showed severe involvement of the gluteal and anterior compartment muscles and all 3 components of the visible hamstring muscles, with clear adductor sparing and marked calf atrophy. This case may represent a more severe and/or more advanced case.

### Muscle Pathology

**Figure 3:**
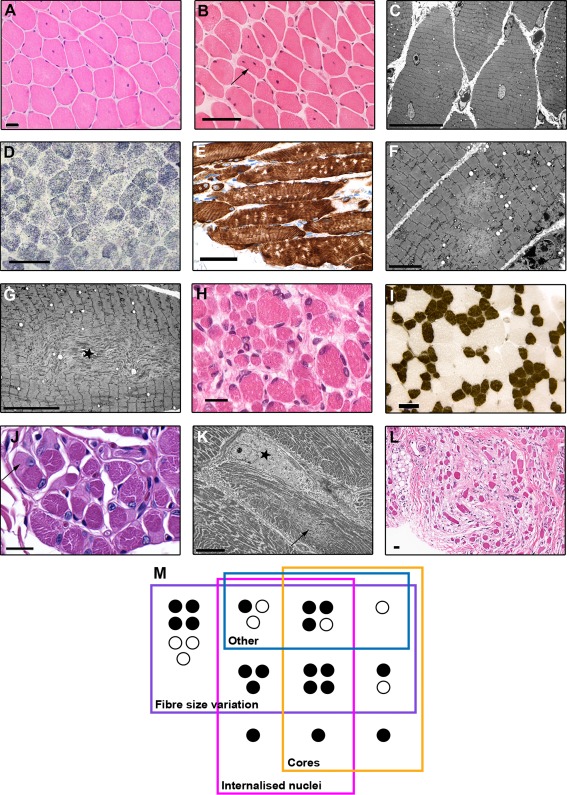
Examples of histopathological and ultrastructural features. All brightfield scale bars = 50 µm unless otherwise stated. (A) Centralized nuclei in the pattern of centronuclear myopathy in a hematoxylin and eosin (H&E)‐stained quadriceps section from the Family 3 female proband at age 5 years. Scale bar = 20 µm. (B) Both internalized and centralized nuclei in an H&E‐stained quadriceps section from the male proband from Family 5, at age 14 years. One fiber *(arrow)* has multiple internalized nuclei, a feature that was much more prominent in biopsies from older patients. This image also shows a very mild increase in endomysial connective tissue. (C) Electron micrograph (EM) image showing internalized nuclei and scattered, small, poorly defined areas of sarcomeric disruption consistent with minicores (Sibling 1/Family 1, quadriceps, age = 14 years, scale bar = 20 µm). (D) Lucent areas compatible with multiminicores in a succinate dehydrogenase–stained section from the same biopsy as shown in C. The multiminicores in D were confirmed ultrastructurally, as shown in F, which presents 2 minicores in a longitudinally orientated fiber (scale bar = 5 µm). (E) Multiminicores as discrete nonstaining foci in a longitudinally orientated paraffin section stained immunohistochemically for desmin (Sibling 2/Family 1, quadriceps, age = 10 years). (G) EM image of a myofiber containing a large centrally placed unstructured core with prominent Z‐band streaming (*star;* Sibling 1/Family 1, quadriceps, age = 14 years, scale bar = 10 µm). (H) Significant fiber size variation in the antemortem quadriceps biopsy taken on day 1, from the male Family 6 proband who died later the same day (scale bar = 20 µm). (I) Classical features of congenital fiber type disproportion, including T1 fibers which are > 25% smaller than T2 fibers, and T1 predominance, in the absence of other abnormalities (adenosine triphosphatase pH 4.3 section from Family 2 proband, site unknown, age = 3 years). (J) Multiple cap‐like regions (one shown with *arrow*) in a periodic acid Schiff–stained paraffin section (Sibling 2/Family 1, quadriceps, age = 10 years, scale bar = 20 µm). (K) EM image of a sharply demarcated cap‐ike region *(star)* characterized by marked myofibrillar disruption, loss of thick filaments, and thickened Z‐discs. The fiber also contains a minicore *(arrow)* and numerous peripheral mitochondria (Sibling 2/Family 1, quadriceps, age = 10 years, scale bar = 10 µm). (L) An area of fibrosis from patient shown in C (H&E, scale bar = 20 µm). (M) Schematic representation of the overlap between the main histopathological patterns seen in patient biopsies. “Other” refers to rarer structural abnormalities: cap‐like regions, ring, coiled, and whorled fibers, and central and peripheral mitochondrial accumulations. Black circles indicate patients with two N2A mutations. White circles indicate patients with 1 N2A mutation and 1 metatranscript‐only mutation.

One patient from each family had undergone at least 1 muscle biopsy (source of data, age, site, and findings are listed in Table 2), with the single exception of Family 19. Examples of biopsy features are shown in Figure 3.

**Table 2:** Summary of skeletal muscle histopathology and ultrastructural (EM) features

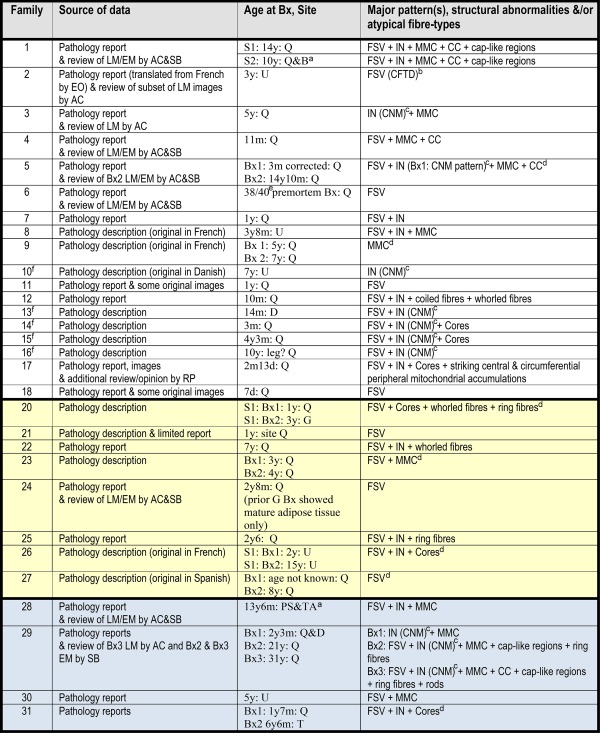

This table summarizes the main histopathological patterns seen in clinical analysis cohort members and in segregation‐inconclusive cases. Families 1–18 had segregation‐confirmed congenital titinopathy. No biopsy was undertaken in the Family 19 proband. Families 20–27 have segregation‐confirmed congenital titinopathy with 1 metatranscript‐only mutation that impacts an exon not included within the reference mature skeletal muscle isoform, N2A. Families 28–31 have a range of congenital titinopathy‐like clinical features (as shown in Supplementary Table 3), but inconclusive segregation results due to absence of carrier status data from one parent (Families 28 and 29) or absence of either mutation in one parent (Families 30 and 31). FSV, IN, cores, and “other” structural features are defined in “Results: Muscle Pathology.” Cases with 1 metatranscript‐only mutation had similar histopathological and ultrastructural findings to other segregation‐confirmed cases, although none had “classical” centronuclear myopathy features. The segregation‐inconclusive case muscle biopsies had similar histopathological features to those in segregation‐confirmed cases.

When no slides were available for review and no EM had been performed, descriptions of both moth‐eaten fibers or central lucencies suggestive of cores on oxidative enzyme stains were recorded as cores.

Similar major features in both biopsies.

Fulfils histopathological diagnostic criteria for CFTD (fiber size variation + type 1 fibers at least 12% smaller than type 2 fibers + type 1 fiber predominance + no additional structural abnormalities; Clarke and North^31^). In this particular case, type 1 fibers were 32% smaller than type 2.

IN pattern consistent with or reported to be consistent with CNM.

Summary of major features in both biopsies from same patient, combined.

38/40 = 38 weeks gestation.

Previously published case (Ceyhan‐Birsoy et al^22^).

ACh = Amanda Charlton (coauthor); B = biceps; Bx = biopsy; CC = centrally placed cores; CFTD = congenital fiber type disproportion; CNM = centronuclear myopathy; D = deltoid; EM = electron microscopy; EO = Emily Oates (coauthor); FSV = significant fiber size variation; G = gastrocnemius; IN = internalized nuclei (considered abnormal if present in ≥3% fibers); LM = light microscopy; MMC = multiminicores; PS = paraspinal; Q = quadriceps; RP = Rahul Phadke (coauthor); S1 = oldest of 2 affected siblings; S2 = youngest of 2 affected siblings; SB = Susan Brammah (coauthor); T = triceps; TA = tibialis anterior; U = site unknown.

All muscle biopsies were abnormal. The histopathologic changes fell into 3 main patterns: (1) increased fiber size variation (FSV; present in ≥ 1 biopsies from 89% of families), (2) increased internalized nuclei (IN; 59%), and (3) cores (48%), with additional structural abnormalities in some cases. Seven biopsies had FSV alone, 1 had IN alone, and 1 had only cores (multiminicores). The remaining biopsies had a combination of ≥2 main patterns with or without additional structural abnormalities (see Table 2, Fig 3M).

Where fiber type information was available, the pattern was of type 1 fiber type disproportion (small predominant type 1 fibers and fiber size disproportion of >12%; see Fig 3H). One biopsy (see Fig 3I; Family 2 proband) fulfilled histopathological criteria for congenital fiber type disproportion.^31^

A subset of biopsies with increased IN (present in ≥ 3% of fibers) had nuclei in the geographical center of the fiber (see Fig 3A,C). Seven biopsies had been given the histopathological diagnosis of centronuclear myopathy (CNM). When the IN were not centralized, and there was significantly increased fibroadipose tissue, fiber splitting, and/or additional architectural abnormalities, the biopsies had typically been reported as “dystrophic” or “severely myopathic” (see Fig 3L). Signs of regeneration and/or degeneration were minimal or absent.

Multiminicores (see Fig 3D–F) were more common than centrally placed cores (see Fig 3G). Centrally placed cores typically had the appearance of larger, perhaps merged minicores. Central cores extending down the majority of the longitudinal fiber axis were not seen on available electron micrograph images.

Rarer structural abnormalities seen (see Table 2) included cap‐like regions (see Fig 3J, K), ring, coiled, and whorled fibers, and striking central and peripheral mitochondrial accumulations. A subset of biopsies showed variable fiber type grouping.

Additional features associated with *MTM1‐*, *DNM2‐* and *RYR1‐*centronuclear myopathy, such as central accumulation of oxidative stains, radial arrangement of sarcomeres, or necklace fibers, were not observed. Rimmed vacuoles (described in HMERF and TMD) were absent.

### Autopsy Findings

The main features of the 2 autopsied deceased patients are shown in Table 3. Brain and spinal cord were normal in both patients.

**Table 3:** Summary of autopsy findings

Family	Cause of Death and Data Source	Autopsy Findings
6	Died on day 1 of life (38/40^a^) due to worsening respiratory distress and poor prognosis. Complete autopsy report and review of LM/EM by ACh & SB.	External: Small for gestational age (weight < 3rd percentile), small placenta, normal fetal/placental ratio, myopathic and dysmorphic facial features, multiple bilateral upper limb contractures (shoulders, elbows, wrists, fingers), reduced palmar creases, bilateral talipes equinovarus, congenital femoral and humeral fractures, thin ribs, undescended testes. Internal (macroscopic and microscopic): Normal brain, spinal cord and heart; pulmonary hypoplasia (lung:body weight ratio = 0.6%; < 1.2% indicates hypoplasia).
10	Died at age 13 yr from pneumonia. Autopsy description (original in Danish).	External: Height and weight < 3rd percentile, retrognathia, muscle wasting, limb contractures (elbows, knees), evidence of previous scoliosis surgery, asymmetrical rib positioning, PEG tube *in situ*. Internal (macroscopic and microscopic): No brain or spinal cord abnormalities, nerve normal; pneumonia, left lower lobe compressed by scoliosis, dilated hypertrophic right ventricle.

a 38 weeks gestation.

ACh = Amanda Charlton (coauthor); EM = electron microscopy; LM = light microscopy; PEG = percutaneous endoscopic gastrostomy tube; SB = Susan Brammah (coauthor).

### Subgroup Analysis Of Patients with Mutations that are predicted to have no Impact on the N2A Skeletal Muscle Isoform Transcript

We compared the clinical features of the 10 patients with metatranscript‐only mutations to those seen in the combined clinical analysis cohort (see Supplementary Table 3). Respiratory involvement appeared less common in this subgroup; however, this subgroup was younger at ascertainment (average age = 9 years vs 12.5 years). Cardiac involvement was also rare (only mild pulmonary stenosis in the Family 25 proband, who also had a DCM‐mutation). The 2 patients with mild intellectual disability and 2 of the 3 patients with structural brain abnormalities were in this subgroup. Internalized nuclei were often present; however, no subgroup member had a centronuclear myopathy biopsy picture. The remaining clinical and histopathological features were similar.

### Cardiac Isoform Analysis

As shown in Table 4, cohort families with 2 mutations predicted to impact both N2BA and N2B cardiac isoforms showed a trend toward an increased likelihood of cardiac involvement (*p*_Fisher_ = 0.087, odds ratio [OR] = 6.0, 95% confidence interval [CI] = 0.78–78). This achieved statistical significance with the inclusion of 5 previously published congenital titinopathy families with 2 truncating mutations (from Carmignac et al,^21^ Chauveau et al,^23^ and Fernandez‐Marmiesse et al^24^) in the analysis (*p* = 0.009, OR = 11, 95% CI = 1.6–130). The pattern held with the addition of the 2 published families with 1 truncating and 1 missense mutation (from Chauveau et al^23^; *p* = 0.002, OR = 13, 95% CI = 2.0–150).

**Table 4:** Cardiac Isoform Analysis

	Cardiac Involvement		Fisher's Exact Test	
Isoform	Yes	No	*p*	OR (95% CI)
Clinical analysis cohort members only				
2 N2BA/N2B^a^	6	2	0.087	6.01 (0.78–78.21)
Other^b^	6	13		
Cohort + published families with 2 truncating muts^21^, ^23^, ^24^				
2 N2BA/N2B^a^	10	2	0.009	10.66 (1.59–129.40)
Other^b^	6	14		
Cohort + published families with 2 truncating muts^21^, ^23^, ^24^ or 1 truncating & 1 missense mut^23^				
2 N2BA/N2B^a^	12	2	0.002	12.75 (1.97–152.30)
Other^b^	6	14		

Table shows the 2 × 2 Fisher's Exact Test tables used to analyze the association between carriage of 2 mutations (*in trans*) that impact both N2BA and N2B cardiac isoforms, and the presence of cardiac pathology in one or more affected family members.

a Patients with 2 mutations predicted to alter both N2BA and N2B cardiac isoforms (see Supplementary Table 1).

b Patients with other combinations of mutations.

CI = confidence interval; mut = mutation; OR = odds ratio.

### Western Blotting

**Figure 4:**
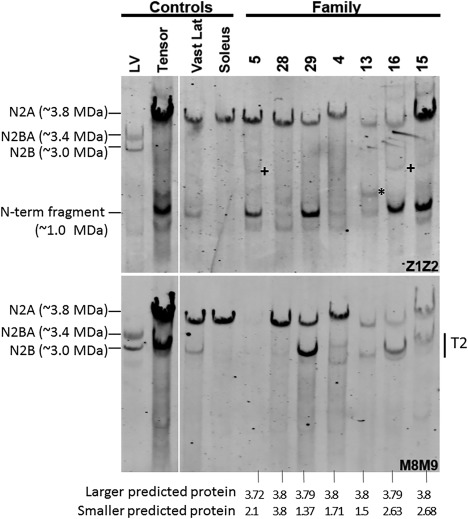
Western blot analysis of patient muscle samples. Antititin antibodies were used that were specific for the titin N‐terminus (Z1Z2; top panel) or C‐terminus (M8M9; bottom panel). The 4 left lanes are controls, and the 7 right lanes are biopsies from patients (from Families 4, 5, 13, 15, and 16), or segregation‐inconclusive cases (Families 28 and 29). All patients have mutations in both of their *TTN* alleles that produce proteins that are predicted to vary in size. The segregation‐inconclusive cases also have 2 *TTN* mutations that are predicted to produce proteins of different sizes if their mutations are (as suspected) *in trans*. The bottom of the figure shows the expected protein mass, assuming that the wild‐type full‐length titin is 3.8MDa and that the mutant protein is reduced by the size of the missing exons(s). The largest predicted proteins are in patient biopsies from Families 28, 29, 4, 13, and 16 at nearly full size (in‐frame deletion of a single small exon near the middle of titin), consistent with their observed expression of a large titin that reacts with both Z1Z2 and M8M9. The largest predicted proteins in the remaining 2 patients (from Families 5 and 15) are slightly smaller than full‐length titin. The patient from Family 5 has a *TTN* truncating mutation in exon 359 reducing its size by 112KDa and eliminating the M8M9 binding site, consistent with the finding that titin in muscle from this patient reacts with Z1Z2 but not M8M9. The patient from Family 15 has a frameshift mutation in exon 364. Although this leaves the binding site for M8M9 intact, Patient 15 titin reacts only weakly with M8M9 (in contrast with the strong Z1Z2 reactivity), suggesting that the antigen availability is reduced, or perhaps that the mutant titin is degraded near its C‐terminus. The second mutant allele is, in all of the patients/cases, predicted to produce a protein that is between 1.5 and 2.7MDa (see bottom of figure) that reacts with Z1Z2 but not M8M9. Although weak bands with the expected reactivity are present in some of the patients, they are very minor relative to (nearly) full‐length titin, except for the band marked by an asterisk in the biopsy from Family 13. Also note that several patients have a Z1Z2‐positive, M8M9‐negative band at a molecular mass of ∼1.0MDa. However, this band is also seen in several control samples and is present at the same size in different patients with different mutations and therefore it is unlikely that this ∼1.0MDa band is mutation‐specific. LV = left ventricle; T2 = degradation product of titin that mainly consists of titin's A‐band segment; Vast Lat = Vastus lateralis.

All 7 muscle biopsies analyzed by western blot showed a band corresponding to full‐length or nearly full‐length titin skeletal muscle isoform; N2A (Fig 4). A full‐length/nearly full‐length band was seen with the N‐terminal antibody in all 7 cases, and with the C‐terminal antibody in 6 of the 7 cases. The absence of predicted truncated proteins in samples with nonsense and frameshifting mutations may be due to nonsense‐mediated decay, or protein degradation eliminating the N‐ and C‐termini. However, western blots with antibodies raised to either titin's I‐band (9D10) or A‐band (MIR) regions did not detect truncated titin proteins (data not shown). An exception is the sample from Family 13 (with 2 splice site changes, 1 of which is predicted to result in in‐frame exon skipping), which contained an additional band that was Z1Z2‐positive but M8M9‐negative (see Fig 4, asterisk). This might represent a truncated protein derived from the mutant *TTN* allele that is predicted to produce a truncated ∼1.5MDa protein. Several other samples contained barely detectable Z1Z2‐positive M8M9‐negative bands (see Fig 4, + symbols) that are not the size of predicted truncation proteins. These might represent mutant titin degradation and/or alternatively spliced products. The reduced band intensity may be due to lower levels of protein production and/or the loss of smaller degradation/splicing products that were not retained on the gels.

### Splice Site Mutation Analysis

A minigene splicing assay showed that the exon 220 c.40558G>C exonic splice site mutation shared by Families 4 and 13 results in in‐frame exon skipping, and that the Family 13 exon 244 c.44816‐1G>A mutation results in frameshift.^22^

cDNA analysis showed that the Family 6 paternally inherited extended splice site deletion resulted in in‐frame skipping of exon 317. No other sample from 70 patients with neuromuscular disease or 184 GTEx (http://gtexportal.org/home/) control adult skeletal muscle samples showed skipping of this exon.

## Discussion

Since MPS “opened the door” to the comprehensive genetic analysis of *TTN*, congenital titinopathy has emerged as an important cause of early onset myopathy. Using MPS, we identified an international cohort of 30 individuals with early onset muscle weakness and/or contractures, and recessively inherited nonsense, frameshift, and/or splice site *TTN* mutations. The clinical features, muscle histopathology, autopsy, and imaging results of cohort members were analyzed to identify features with clinical and/or diagnostic utility.

One of the most striking findings was the degree of axial involvement, which was present in all patients, manifesting as one or more of the following features: neck weakness, early onset progressive scoliosis, chest wall deformities and early onset respiratory insufficiency. Axial complications, including scoliosis and respiratory impairment, typically progressed rapidly, whereas limb weakness was often stable or only slowly progressive, and insufficient to prevent acquisition of independent walking in the majority of patients. This truncal‐predominant phenotype is reminiscent of *SEPN1‐* and severe nemaline myopathy, and *COL6‐*, *LAMA2‐* and *LMNA*‐congenital muscular dystrophy.^32^, ^33^ Congenital contractures were also common, affected distal as well as proximal joints, and often involved two or more areas of the body, leading to the diagnosis of AMC. Acquired limb contractures were common, as was joint hypermobility, features shared with *COL6*‐myopathy.^33^

Respiratory complications were the most common cause of death. Progressive respiratory insufficiency typically developed within the first 12 years of life, often necessitating assisted ventilation. With 1 exception, respiratory insufficiency occurred in patients with concomitant spinal and/or chest wall deformities. Many patients with significant respiratory impairment remained ambulant, a feature also shared with early onset *SEPN1‐*, *NEB‐*, *TPM3‐ ACTA1*‐myopathy and *COL6*‐muscular dystrophy.^32^

Almost 50% of patients had congenital or early onset cardiac abnormalities. Cardiomyopathy is sometimes associated with muscle disorders caused by *MYH7, LMNA, FKRP, FKTN, SPEG, ACTA1* and *TAZ* mutations^32^, ^33^, ^34^, ^35^; however, it is not typically associated with other forms of early onset muscle disease. Congenital cardiac defects and a history of dilated cardiomyopathy in heterozygous relatives are not typical features of other congenital myopathies. Congenital titinopathy should be considered in the differential diagnosis of patients with any of these cardiac/family history features.

It has recently been shown that heterozygous *TTN* truncating and essential splice site mutations that alter both N2BA and N2B, the 2 longest and most abundant adult cardiac isoforms, increase the risk of adult onset DCM.^15^ Truncating mutations that alter fetal and/or the smaller novex cardiac isoforms, or that impact only the N2BA isoform (N2BA‐only mutations) are not associated with DCM in the heterozygous state.^16^ This is presumably because the predominant adult cardiac isoform (N2B) can still be transcribed from the truncating allele. Missense variation in *TTN* is common, and although some individual variants cause cardiomyopathy, it is not currently possible to interpret the DCM risk associated with the vast majority of these variants.

Our data suggest that congenital titinopathy patients with 2 mutations predicted to impact both N2BA and N2B cardiac isoforms (N2BA/N2B mutations) are significantly more likely to have cardiac involvement than those with other combinations of mutations. With the available cohort and previously published cases, there was insufficient power to determine whether patients carrying 1 N2BA/N2B mutation are at higher cardiac risk than those with biallelic N2BA‐only mutations (ie, 2 mutations that alter N2BA but spare N2B), as would be expected by extrapolating from the *TTN* DCM literature. There were also insufficient data to confirm whether the pattern of familial risk is highest for relatives with N2BA/N2B mutations, although both families with a maternal family history of DCM had a maternal N2BA/N2B mutation.

If it is confirmed that having biallelic N2BA/N2B mutations confers the highest risk of cardiac involvement in congenital titinopathy, this might explain why the cohort members with 1 metatranscript‐only mutation (all of which spare N2BA and N2B) had fewer cardiac abnormalities than other patients (only 1/10 had a cardiac anomaly: mild pulmonary stenosis).

Until the cardiac risk factors associated with this disorder are better understood, and the association between risk and cardiac isoform involvement is confirmed, cardiac screening is strongly recommended for all congenital titinopathy patients, and should be considered in heterozygous carrier relatives, particularly those carrying truncating or splice‐altering variants in cardiac constitutive exons.

In combination, the muscle biopsy results of this study confirm that congenital titinopathy is a pathological “chameleon,” presenting with a wide range of structural abnormalities. Central/internalized nuclei and cores have been reported in other congenital titinopathy cases^21^, ^22^, ^23^; however, a typical congenital fiber type disproportion muscle picture, cap‐like regions, rods, ring, whorled, and coiled fibers, and central and circumferential peripheral mitochondrial accumulations have not been reported previously. Overall, fiber size variation, cores, and internalized nuclei, either alone, or in combination, were the most common histopathological abnormalities.

Muscle from congenital titinopathy patients sometimes showed a typical CNM pattern. The absence of ophthalmoplegia may be helpful in discriminating between congenital titinopathy and other CNM genetic subtypes.

A subset of muscle biopsies had a dystrophic appearance, sometimes in association with elevated CK, suggesting histopathological overlap with congenital muscular dystrophies. Congenital titinopathy is also an important diagnosis to consider in genetically unresolved core and cap myopathy cases.

Ten patients from 8 families had 1 mutation that spares the N2A skeletal muscle isoform: a metatranscript‐only mutation. These mutations were within 1 of 4 exons (163, 172, 181, and 201) included within the I‐region PEVK segment of the established complete *TTN* metatranscript. In further support of the pathogenicity of these mutations, 1 of these mutations (exon 163: c.35794G>T; p.Glu11932*) was shared by 5 unrelated cohort families and 1 segregation‐inconclusive case. The utility of a subgroup analysis of clinical features associated with patients carrying 1 metatranscript‐only mutation was limited given the small subgroup size; however, the clinical features were largely similar to other cohort members. Furthermore, Fernandez‐Marmiesse et al recently described an AMC case with typical congenital titinopathy clinical and histopathological features (without cardiac involvement), and a homozygous truncating mutation within an additional metatranscript‐only exon.^24^ Together, these findings suggest that the metatranscript‐only mutations identified in our cohort patients are “true” congenital titinopathy‐causing mutations.

Titin developmental isoforms have been characterized in multiple species, including humans, mice, and rabbits.^9^, ^10^ They are longer than their mature muscle counterparts, due to an elongated I‐band PEVK spring segment that includes exons absent from mature isoform transcripts. Human fetal skeletal muscle isoforms have not been formally characterized; however, 3 of the 4 metatranscript‐only PEVK exons (163, 172, and 181) were originally detected in a human fetal skeletal muscle cDNA expression library,^36^ and 3 (163, 172, and 201) have been detected in mouse fetal skeletal muscle.^10^ Overall, these findings suggest that the pathogenesis of congenital titinopathy can result from mutations in exons that are present in one or more developmental isoform(s).

All muscle samples analyzed by western blot showed full‐length or nearly full‐length titin. This finding raises the possibility that the presence of a near‐normal sized protein might be essential to survival. This is supported by mouse studies that show that *Ttn*‐deficient mice are embryonic lethal due to abnormal cardiac morphogenesis and dysfunction.^37^, ^38^

Interestingly, all families had at least 1 mutation predicted to result in production of a near‐normal sized titin. Most had at least 1 C‐terminal M‐line exon mutation, or at least 1 splice site mutation predicted or shown to cause in‐frame loss of a single exon, which should result in a near‐normal protein product. All remaining patients had 1 metatranscript‐only mutation whose impact is unknown but might not affect transcript length or protein size in mature muscle. Some natural readthrough of nonsense mutations might also be responsible for minor amounts of full‐length protein seen on western blot. Protein analysis of muscle from additional patients will shed further light on whether the presence of a full, or near full‐length protein band is a consistent finding in patients who do not succumb to the disorder during development.

Congenital titinopathy increasingly appears an important, common, and potentially severe form of axial‐predominant congenital myopathy. Analysis of the clinical, histopathological, imaging, and autopsy features of this 30‐member truncating/splice mutation cohort significantly expands our understanding of the clinical features and natural history of this disorder. This study will facilitate the diagnosis and management of affected individuals, inform cardiac surveillance in heterozygous relatives, and guide clinical decision making around severely affected infants. In addition, the unexpected discovery of metatranscript‐only mutations in a significant subset of patients suggests that yet‐to‐be‐characterized developmental skeletal muscle isoform(s) are involved in the pathogenesis of this disorder. Studies are currently underway to further improve cardiac risk prediction, gain a molecular‐level understanding of disease‐associated mutations at the RNA and protein levels, and extend our understanding of the pathological mechanisms involved in this emerging disorder.

## Author Contributions

E.C.O., K.J.J., A.Ch., S.B., J.S.W., K.S.Y., I.R., B.U., A.F., K.N.N., N.F.C., M.L., A.H.B., C.G.B., D.G.M., H.G., M.R.D., and N.G.L. were responsible of study concept and design. E.C.O., K.J.J., S.D., A.Ch., S.B., J.E.S., J.S.W., K.S.Y., N.Wh., M.A.F., I.R., S.T.C., B.U., A.F., N.F.C., A.H.B., M.M.R., C.G.B., H.G., M.R.D., and N.G.L. were responsible for drafting the manuscript and the figures. E.C.O., K.J.J., S.D., A.Ch., S.B., J.E.S., J.S.W., K.S.Y., L.C.S., N.Wh., A.J.P., A.B., L.B.W., M.A.F., H.A.S., H.L.T., P.J.L., D.M., R.B.F., A.J.C., M.M.R., G.L.O., S.A.S., R.G., H.J., J.L.M., M.A.N., S.K., J.P., A.T., E.H., M.B., C.A.G., M.M., U.W., N.S., A.P., I.M., J.R.P., C.V., C.G., A.Ca., P.M., M.E.L., A.R.F., D.B.‐G., J.C., A.M.C., H.R.G., S.T.I., D.C., B.B.C., R.I.W., L.L., J.V., S.C., N.D., H.‐M.L., N.H.T., N.C.F., M.A.I., S.E., C.A.M., R.P., G.R., N.Wi., P.H., I.R., S.T.C., E.‐J.K., E.P.H., K.B., V.S., B.U., A.F., N.F.C., M.L., A.H.B., C.G.B., D.G.M., H.G., and M.R.D. were responsible for data acquisition and analysis. All authors read and approved the final version of the manuscript.

## Potential Conflicts of Interest

Nothing to report.

## Supporting information

Additional supporting information may be found online in the Supporting Information section at the end of the article.

Supporting Information

Supporting Information

Supporting Information

Supporting Information
