# Supplementary material for: Congenital Titinopathy: Comprehensive characterization and pathogenic insights
Source: Ann Neurol. 2018 Jul 27;83(6):1105–24. doi: 10.1002/ana.25241 (PMC6105519; doi:10.1002/ana.25241)
Supplement: Supplementary file 1 — Supporting Information [file ANA-83-1105-s001.docx]

**Supplementary tables**

- **Supplementary Table 1: Comprehensive summary of mutation and cardiac data**
- **Supplementary Table 2: Comprehensive summary of clinical features**
- **Supplementary Table 3: Comparison of clinical features in cases with one “metatranscript-only” mutation with features seen in overall cohort**

(note: features of four segregation-inconclusive cases are also compared to clinical analysis cohort members)
