# Supplementary material for: Congenital Titinopathy: Comprehensive characterization and pathogenic insights
Source: Ann Neurol. 2018 Jul 27;83(6):1105–24. doi: 10.1002/ana.25241 (PMC6105519; doi:10.1002/ana.25241)
Supplement: Supplementary file 3 — Supporting Information [file ANA-83-1105-s003.docx]

| **Demographic**  **or clinical feature** | **Percentage of clinical analysis cohort members with feature**  **&/or additional descriptive data** |
| --- | --- |
|  |  |
|  |  |
| **Description of families/cohort members included in clinical analysis** | **30 affected individuals from 27 families**:10 females, 20 males  3 sibling pairs (Families 1, 20, 26)  5 deceased individuals (Families 1 (Sibling 2), 6, 7, 10, 18)  (autopsy data on 2 of the 5 deceased individuals: shown in **Table 3**)  4 cases published previously (Families 13-16: Numbered BOS314-1, BOS979-1, BOS1044-1 and BOS 1093-1 in original publication by Ceyhan-Birsoy et al [22]) |
| **Age at ascertainment**  **or age at which affected individual became deceased**  (based on information provided for all 30/30 cohort members) | Living cohort members were aged 9m to 34y:  Ages of 25 living cohort members:  **0 to 5 years** = 7 (28 % of living members)  (9m, 2y3m, 2y5m, 2y6m, 3y9m, 4y3m, 5y)  **6-12 years** = 7 (28 %)  (6y, 6y, 6y, 7y, 8y, 10y, 11y)  **13-17 years** = 6 (24 %)  (13y, 13y, 14y, 14y, 14y, 15y)  **18 years or older** = 5 (20 %)  (18y, 19y, 21y, 32y, 34y)  Age at time of death for 5 deceased cohort members = 38/40 (day 1 of life), 3m, 8y, 13y, early 30s |
| **Parental ancestry/consanguinity**  (based on information provided for all 27/27 families) | Diverse ancestry  24 non-consanguineous families: 2 consanguineous families (Families 2 & 7)  1 likely consanguineous (Family 18: both parents from same valley & share same mutation) |
| **Family history of neuromuscular or cardiac disorders / abnormalities?**  (based on information provided for all 27/27 families) | **No history of neuromuscular disorders** or symptoms/signs in first degree family members  (Including no TMD features in maternal parent with TMD mutation from BOS 1044-1 = Family 15)  **History of cardiac abnormalities in 3/27 families** (11%)  Family 12: Prior pregnancy terminated due to congenital heart disease  Family 13: Mother of affected individual, maternal grandmother & maternal aunt have subclinical cardiomyopathy: all three confirmed heterozygous carriers  Family 9: = Maternal aunt of affected individual has cardiomyopathy: carrier status not known  **Note:** Many carrier-confirmed parents and siblings had not had cardiac screening at time of ascertainment |
| **Onset** |  |
| **Age at onset / age when symptoms/signs first noted**  (based on information provided for all 30/30 cohort members) | *In utero* = 17 cases (57%)  Birth = 9 (30%)  Infancy = 4 (13%) (symptoms and/or signs first noted at 3m corrected, 3m, 8m, and 14m) |
| **Pregnancy/birth history** |  |
| **Reduced fetal movements**  (based on information provided for 29/30 cohort members) | 17/29 (59%)  (data not available for one cohort member) |
| **Abnormal liquor volume?**  (based on information provided for 23/30 cohort members) | 4/23 (17%)  Amniotic infusion required at 30/40 for 1 case |
| **Additional *in utero* ultrasound anomalies**  (based on information provided for 27/30 cohort members)  Note: IUGR = intrauterine growth retardation | 7/27 (26%)  Description:   1. IUGR in 3 cases (1^st^ noted 32/40, 27/40; gestation first noted not available for 3rd: growth hormone deficiency later detected in 1 of these 3 cases) 2. Limb contractures in 1 case 3. Increased nuchal thickness on 12/40 scan in 1 case: additional talipes noted on 15/40 and 20/40 scans 4. Dilated stomach on 33/40 and 36/40 scans in 1 severely affected case 5. Left hydronephrosis at 32/40: no longer present on neonatal ultrasound with normally functioning kidneys |
| **Gestation at delivery**  (based on information provided for 28/30 cohort members) | Preterm (less than 37 weeks gestation) = 3/28 (11%)  (age at delivery: 34/40, 35+6/40, 36+6/40)  Term (at or after 37 weeks gestation) = 25/28 (89%) |
| **Abnormal presentation at delivery?**  (based on information provided for 27/30 cohort members) | Normal presentation = 20/27 (74%)  Breech = 6/27 (22%)  Vertex compound presentation with both hands positioned on sides of head = 1/27 (4%) |
| **Congenital features (excluding cardiac)** |  |
| **Congenital hypotonia/weakness**  (based on information provided for 27/30 cohort members) | 19/27 (70%) |
| **Congenital limb contractures**  (based on information provided for 28/30 cohort members) | 17/28 (61%)  Location:   1. Finger (fixed flexion or extension or mixed)/hand = 9 2. Wrist = 7 3. Elbows = 6 4. Congenital dysplasia of hips = 2 5. Hip = 2 6. Knees = 3 7. Ankle contractures/Talipes = 9 (talipes calcaneovalgus = 1, talipes equinovarus= 3, type not specified = 5)   Note: congenital contractures typically bilateral: unilateral in a small subset of cases  Congenital contractures involving two or more body areas (“arthrogryposis multiplex congenita”) in at least 12 cases: 12/28 (43%) |
| **Congenital scoliosis**  (based on information provided for 26/30 cohort members) | 2/26 (8%)  (Note: both cases were siblings from Family 1) |
| **Congenital fractures**  (based on information provided for 27/30 cohort members) | 2/27 (7%) |
| **Neonatal respiratory difficulties**  (based on information provided for 29/30 cohort members) | 12/29 (41%)  Description:   1. Required intubation and ventilation during neonatal period = 4/29 (14%) (1^st^ died at 9 hours of age due to profound respiratory failure / decision to withdraw support, 2^nd^ died from respiratory failure at 3m due to failure to improve / decision to withdraw care, 3^rd^ still requires full time ventilatory support via tracheostomy at 9m, 4^th^ only required a short period of intensive support after birth) 2. Dependent on non-invasive ventilation since birth = 1 3. Required brief period of non-invasive respiratory support = 4/29 (14%) 4. Needed respiratory assistance in neonatal period in context of Pierre Robin Sequence = 1 5. Supplemental oxygen requirement only = 1 6. Required respiratory support: type not specified = 1 |
| **Neonatal feeding difficulties**  (based on information provided for 27/29 cohort members) | 17/27 (63%)  Description: Poor suck in all cases with difficulties: a subset required nasogastric tube feeding |
| **One or more additional congenital features**  (based on information provided for 23/30 cohort members) | 13/23 (57%)  Description:   1. Torticollis = 2 2. Reduced or abnormal palmar creases: at least 3 3. Undescended testes = 2 4. Submucous cleft palate = 2 5. Pierre Robin Sequence with cleft = 1 6. Neck webbing with low posterior hairline = 1 7. Bilateral axillary pterygium = 1 8. High thumb insertion site = 1 9. Asymmetrical limb movements (moved right limbs more than left) = 1 10. Hypoglycaemia = at least 1 11. Growth hormone deficiency = 1 12. Several cases with additional non-myopathic facial features described in “Facial features” section 13. Additional features found at autopsy including small size for gestational age, thin ribs and long bones, pulmonary hypoplasia and bilateral pleural effusions (see **Table 3**) = 1   Note 1: A subset of cohort members had more than one of the above features  Note 2: Resuscitation at birth was difficult in at least one case. This baby underwent therapeutic cooling, developed subsequent self-limiting seizures (settled by 48 hours) and showed early signs if hypoxic ischaemic encephalopathy on head MRI |
| **Early motor development and ambulation** |  |
| **Age able to sit independently**  (based on information provided for 20/28 cohort members who survived > 3 months) | Normal age at acquisition (9 months or less) = 8/20 (40%)  Delayed (> 9m) = 11/20 (55%)  (age at acquisition: > 9m, 10m, 10m, > 10m, 11m, 15-18m, 18m, 21m, 2y, 2y; delayed but ? age)  Possibly delayed (“>8m”) = 1 |
| **Ambulation / Rate of loss of ambulation**  (based on information provided for 27/27cohort members who survived > 12 months)  *Rate of loss of ambulation:   - Stable or slow defined as still ambulant or likely to be still ambulant after 20 years of age, - Moderate defined as loss or likely loss of ambulation between 10 and 20 years of age - Rapid defined as loss or likely loss of ambulation before 10 years of age | Number of cohort members who can or were able to ambulate independently = 19/27 (70%)  Number of cohort members who are less than five years of age and have not yet achieved independent ambulation but are continuing to gain motor skills = 3/27 (11%)  (age at study ascertainment: 2y3m, 2y6m, 3y9m)  Number of cohort members aged five years or more who have never been able ambulate independently, and are unlikely to achieve this ability = 5/27 (19%)  (age at study ascertainment: 5y, 6y, 9y, 14y, 19y)  Of the 19 cohort members who can or were able to ambulate independently (without calipers or other equipment):  Walking and still gaining rather than losing limb strength / ambulatory ability = 4/19 (21%)  Stable or slow* progression in limb weakness / loss of ambulatory ability = 9/19 (47%)  Moderate* = 4/19 (21%)  Rapid* = 1/19 (5%)  No limb weakness (aged 21y) = 1/19 (5%)  Note: One additional cohort member was able to ambulate with calipers initially but became wheelchair dependent by age 4y due to contractures and progressive weakness. Now, at age 18: he uses combination of power and manual wheelchair; finding transfers increasingly difficult. This case was Included in “unable to ambulate independently” category |
| **Age first able to walk independently**  (based on information provided for 19/19 cohort members or can or did achieve independent ambulation) | Of the 19 cohort members who can or were able to ambulate independently:  Non-delayed age at first walking (ambulation achieved at or before 18 months of age) = 10/19 (53%)  (age at acquisition: 11m, 13m, 15m, 15-18m, 15-18m, 16m, 16m, 18m, 18m, 18m)  Delayed walking (ambulation achieved after 18m) = 9/19 (47%)  (22m, 2y2m, 2y3m, 3y, 3y, 3y, 5y, 6y, “late”) |
| **Still walking?**  (based on information provided for 19/19 cohort members or can or did achieve independent ambulation) | Number of cohort members who have maintained independent ambulation, once attained = 18/19 (95%)  Number of cohort members who have lost ambulation = 1/19 (5%) (lost ability at age 7y: cause = progressive contractures and foot deformities)  Number of cohort members who are having marked difficulties with ambulation = 3/19 (16%)  (due to increasing fatigability, weakness, contractures, pain on exertion) |
| **Of those able to walk: ever able to run?** | At least 8/19 (42%) (slow jog for some, fast run for others) |
| **Ever able to walk fast but not run?** | At least 1/19 (5%) |
| **Ever able to jump?** | At least 4/19 (21%)  (from age 4y, 5y: age at acquisition not documented for 3^rd^ or 4^th^ cohort members) |
| **Most recent gait description** | Examples of descriptive terms used to describe gait in cohort members:  “slow gait, waddling gait, asymmetrical gait due to scoliosis, Trendelenberg gait, tendency to crouch, flat footed gait, in-toeing gait, hyperlordotic, feet abnormally placed when walking, foot drop, walks with hyperextended knees, walks with genu valgus deformities,”  Normal gait also described in a subset of cases  Frequent falls mentioned often |
| **Gowers’ positive?** | Of those 19 cohort members who can or were able to ambulate independently  11/19 (58%) described as currently or previously (at some stage in the past) having a positive Gowers’ manoeuvre.  1 case had a Gowers’ manoeuvre from 3-6y, but had lost it by age 7y |
| **Pattern of limb weakness** |  |
| **UL predominant, LL predominant or UL&LL involvement?**  (based on information provided for 23/28 cohort members who survived past day 1 and had limb weakness) | UL & LL = 15/23 (65%)  UL predominant = 3/23(13%)  LL predominant = 5/23 (22%)  Note1: UL predominant contractures at birth/autopsy in 1 additional case (this case not included in 28 overall cases for this feature as pattern of weakness not reported and child very unwell at birth > died on day 1)  Note2: 1 case had no limb weakness at age 21; also not included in 28 overall cases for this item |
| **P = Proximal, D = Distal**  **P & D = proximal and distal muscles involved approximately equally: P > D = proximal predominant: D > P = distal predominant**  (based on information provided for 25/28 cohort members who survived past day 1 and had limb weakness) | P & D = 6/25 (24%)  P > D = 12/25 (48%) (slight difference only in at least 1 case: P only in one case)  D > P = 5/25 (20%)  Other = 2/25 (8%) (1 case = P > D in UL but D > P in LL: 1 case = P > D in UL but P & D in LL) |
| **Severity of limb weakness**  (based on information provided for 26/28 cohort members who survived past day 1 and had limb weakness) | Mild (MRC grade generally 4/5 or more) = 3/26 (12%)  Mild to moderate (MRC grade generally 3 to 4/5) = 15/26 (58%)  Moderate (MRC grade generally 3/5) = 3/26 (12%)  Mild to severe (MRC grade generally 2/5 to 4/5) = 2/26 (8%)  Moderate to severe (MRC grade generally 2/5 to 3/5) = 1/26 (4%)  Severe (MRC grade generally 2/5 or less) = 2/26 (8%) |
| **Symmetrical or asymmetrical**  (based on information provided for 22/28 cohort members who survived past day 1 and had limb weakness) | Symmetrical = 15/22 (68%)  Asymmetrical = 7/22 (32%) |
| **Deep tendon reflexes (DTR)**  (based on information provided for 21/29 cohort members who survived past day 1) | Normal 1/21 (5%)  Reduced/trace 6/21 (29%)  Absent = 11/21 (52%)  Mixed picture: some absent / some reduced = 3/21 (14%)  At least 3 cohort members: DTR originally present at birth but lost over time (by 5 years in 1 case)  Plantar responses normal in at least 6 cases: no information available re: this feature for remaining cohort members |
| **Muscle bulk** |  |
| **Pattern of muscle hypotrophy**  (based on information provided for 20/29 cohort members who survived past day 1) | Generalised hypotrophy = 15/20 (75%)  Distal hypotrophy (wasting limited to the hands and feet) = 1/20 (5%)  No hypotrophy = 4/20 (20%)  More specific descriptions provided in a subset of cases:  1 = Specific mention of deltoid and lower leg hypotrophy  1 = specific mention of bilateral temporal muscle hypotrophy  1 = Generalised reduction in muscle bulk with tapered lower legs  1 = Hypotrophy of shoulder girdle muscles, biceps and triceps muscles in upper limbs & thigh posterior compartment and tibialis anterior muscles in lower limbs |
| **Muscle hypertrophy / pseudohypertrophy**  (based on information provided for 21/29 cohort members who survived past day 1) | 1/21: calf hypertrophy (Family 13 proband) |
| **Non-congenital (acquired) contractures** |  |
| **One or more non-congenital (acquired) limb contractures**  (based on information provided for 24/28 cohort members who survived > 3 months) | 16/24 (67%) |
| **Two or more non-congenital limb contractures**  (based on information provided for 22/28 cohort members who survived > 3 months) | 10/22 (45%) |
| **Ankle (tendoAchilles)**  (based on information provided for 22/28 cohort members who survived > 3 months) | 8/22 (36%)  Bilateral in the majority of cases. |
| **Knee**  (based on information provided for 22/28 cohort members who survived > 3 months) | 6/22 (27%)  Mild to severe: 1 after femur break |
| **Hip**  (based on information provided for 22/28 cohort members who survived > 3 months) | 6/22 (27%)  Mild to severe  At least 1 required hip osteotomy |
| **Shoulder**  (based on information provided for 22/28 cohort members who survived > 3 months) | 2/22 (9%)  First noted at age 4y6m and age 19y |
| **Elbow**  (based on information provided for 24/28 cohort members who survived > 3 months) | 5/24 (21%)  1 had congenital elbow contractures that resolved with physiotherapy but reappeared later |
| **Wrist/Hand/Finger**  (based on information provided for 24/28 cohort members who survived > 3 months) | 5/24 (21%)  Various combinations of finger flexor and extensor involvement described |
| **Facial features** |  |
| **Facial weakness**  (based on information provided for 27/29 cohort member who survived > 1 day) | 19/27 (70%)  Mild to moderate in almost all cases (only 1 = described as moderate to severe)  Lower face only in one case  Asymmetrical in at least 1 case |
| **Ptosis**  (based on information provided for 21/29 cohort members who survived > 1 day) | 7/21 (33%)  Typically mild, non-fluctuating, usually bilateral  Asymmetrical in at least 1 case  Congenital with diurnal variation in severity in at least 1 case. |
| **Ophthalmoplegia**  (based on information provided for 26/29 cohort members who survived > 1 day) | Absent in all 26/26 cases (100%) for which data available. |
| **High arched palate**  (based on information provided for 26/29 cohort members who survived > 1 day) | 19/26 (73%)  Unable to determine because of Pierre Robin Sequence cleft in 1 case |
| **Additional facial features** | One or more additional facial features in **18** cohort members  Description:  Myopathic features:   1. Long or elongated face = 5 (1 as isolated facial anomaly, 2 in combination with other facial anomalies) 2. Tented upper lip with open mouth posture = 1   Non-myopathic dysmorphic facial features:   1. Asymmetry in subcutaneous facial tissue / tissue bulk = 3 2. Retrognathia (in context of PRS in one) = 2 3. Micrognathia = 1 4. Short palpebral fissures = 2 5. Down-slanting palpebral fissures = 1 6. Small mouth / reduced mouth width = 2 7. Plagiocephaly = 2 8. Dolichocephaly = 1 9. Facial hypoplasia = 1 10. Midfacial hypoplasia = 1 11. Hypotelorism = 1 12. Shallow sagittal nose tip groove = 1 13. Reduced pinna length = 1 14. Dental malocclusion = 1 15. Intermittent right esotropia = 1 16. Mild macroglossia = 1 17. Dysmorphic features not otherwise specified = 1 |
| **Vocal cord/bulbar/pharyngeal involvement** |  |
| **Ongoing chewing/swallowing difficulties**  (based on information provided for 21//28 cohort members who survived > 3 months) | 8/21 (38%) cohort members  Description (Note: some cases had more than one difficulty described):   1. Slow chewing/eating = 2 2. Ongoing sucking difficulties = 1 3. Difficulties with chewing = 1 4. Struggles to eat difficult textures e.g. meat and cereal = 1 5. Cannot eat solids = 1 6. Morning coughing thought secondary to gastrooesphageal reflux = 1 7. Orosensory abnormalities = 1 8. Oromotor dysphagia = 1 9. History of aspiration from young age = 1 |
| **Current requirement for nasogastric tube (NGT) and/or perenteral gastrostomy (PEG) feeding?**  (based on information provided for 27/28 cohort members who survived > day 1 and are still alive) | 7/27 (26%)  Description:   1. Required nasogastric tube feeding for several years but now orally fed = 1 2. PEG now only used for hydration during times of acute illness or refusal of oral supplements = 1 3. NGT tube fed since age 8. Able to eat orally, but requires supplemental feeds via NGT = 1 4. Combined oral and supplemental NGT feeding since infancy (PEG placed at 3 months)   Note: 1 additional cohort member: required NGT feeds for several years, but now orally fed (therefore not included in 6 “yes” cases) |
| **Voice / vocal cord abnormalities** | Nasal voice = 2  Low volume voice = 1  Laryngomalacia = 1 |
| **Neck features** |  |
| **Neck flexion weakness**  (based on information provided for 21/29 cohort members who survived > 1 day) | 20/21 (95%) (exception; the single non-weak Family 19 proband)  MRC grade 2/5 to 4/5  At least 1 case: neck flexion weaker than extension |
| **Neck extension weakness**  (based on information provided for 18/29 cohort members who survived > 1 day) | 15/18 (83%)  MRC grade 2/5 to 4/5  3 cases reported to have a “dropped head” phenotype suggestive of marked neck extension weakness |
| **Limited range of neck movement**  (based on information provided for 18/29 cohort members who survived > 1 day) | 3/18 (17%)  (Note: neck pain reported in a subset of cases with or without limited range of movement) |
| **Axial features and respiratory insufficiency** |  |
| **Scoliosis**  (based on information provided for 28/28 cohort members who survived > 3 months) | 16/28 (57%)  Includes 2 cases from same sibship with congenital scoliosis that progressed significantly after birth  Progressive in all cases: rapidly progressive in most cases.  Brace, surgery or both needed in a significant subset of cases |
| **Kyphosis**  (based on information provided for 24/28 cohort members who survived > 3 months) | 3/24 (13%) |
| **Spinal rigidity**  (based on information provided for 22/28 cohort members who survived > 3 months) | 6/22 (27%)  (Note: spinal fusion surgery in a subset of cases: therefore not possible to assess for this feature) |
| **Hyperlordosis**  (based on information provided for 21/28 cohort members who survived > 3 months) | 6/21 (29%) |
| **Chest wall deformity?**  (based on information provided for 23/28 cohort members who survived > 3 months) | 10/23 (43%)  Most cases pectus excavatum  In a small subset of additional cases rotational deformity of spine had resulted in prominence of one side of chest wall: counted as a “no” for chest wall deformity as secondary to spinal deformity |
| **Scapular winging**  (based on information provided for 20/28 cohort members who survived > 3 months) | 9/20 (45%) |
| **Objective evidence of respiratory insufficiency (abnormal FVC and/or oxygen and/or ventilation requirement)**  (based on information provided for 27/29 cohort members who survived > day 1) | 17/27 (63%) |
| **Correlation between spinal and/or chest wall abnormalities and respiratory insufficiency?**  (based on information provided for 28/28 cohort members who survived > 3 months) | Respiratory insufficiency alone = 2/28 (7%)  (Includes a 9 month old baby on full time ventilation. Other case is 32 years of age who was lost to follow-up from age 17)  Scoliosis alone = 3/28 (11%)  Chest wall abnormality alone = 3/28 (11%)  Scoliosis and chest wall abnormality but no respiratory involvement = 1/28 (4%)  Respiratory insufficiency and scoliosis but no chest wall deformity = 8/28 (29%)  Respiratory insufficiency and chest wall abnormality but no scoliosis = 2/28 (7%)  Respiratory insufficiency and scoliosis and chest wall abnormality = 4/28 (14%)  At least one of these features (scoliosis, chest wall deformity, or respiratory insufficiency) present = 23/28 (82%)  None of these features present = 5/28 (18%) |
| **Other respiratory abnormalities** |  |
| **Frequent /severe respiratory infections?**  (based on information provided for 25/28 cohort members who survived > 3 months) | 5/25 (20%)  Descriptions:   1. 7 episodes of pneumonia before age 2y6m = 1 2. Recurrent pneumonia in infancy, presumed to be caused by recurrent aspiration however frequent infections continued after oral feeding ceased resulting in bronchiectasis = 1 3. One or more severe childhood respiratory infections requiring hospitalisation = 2 4. Frequent/severe respiratory infections not otherwise defined = 1 |
| **Weak cough?**  (based on information provided for 18/28 cohort members who survived > 3 months) | 8/18 (44%)  Cough assist machine required daily or during respiratory illnesses in a subset of cases |
| **Most FVC recent measurement as % of normal (age at Ax)** | Examples of abnormal FVC measurements provided for 3 cohort members:  52% at almost 6y, 77% at age 7y, 44% at 19y  Many cohort members too young for FVC measurement or FVC data not available |
| **Nocturnal or full time ventilation requirement?**  (based on information provided for 25/29 cohort members who survived > day 1) | 11/25 (44%)  1 case was prescribed nocturnal ventilation but unable to tolerate it (still counted as “yes”)  1 case continues to be reliant on full time ventilatory support via tracheostomy at 9 months of age (started: birth)  1 case was reliant on full time ventilatory support until care was withdrawn at 3 months of age (started: birth)  I case has been reliant on nocturnal CPAP from birth  1 case is borderline for need at age 16 years: not included in “yes” cases |
| **Age nocturnal or full time ventilation commenced**  (based on information provided for all 11/11 cohort members who require nocturnal or full time ventilatory support and who survived > 1 day) | 1. From birth = 3/11 (27%) 2. Infancy 3. 18m 4. 2y6m 5. 2y6m 6. 5-6 y 7. 13y 8. 13 years (following out of hospital respiratory arrest) 9. Late childhood (following a respiratory arrest at time of scoliosis surgery; sibling of case who had an out of hospital respiratory arrest) |
| **Diaphragmatic / paradoxical breathing pattern?**  (based on information provided for 12/27 cohort members who survived beyond day 1 and did not require full time ventilatory support) | 2/12 (17%)  Note: very difficult to tell if this feature was present in infants requiring full time ventilatory support |
| **Cardiac abnormalities (including congenital)** |  |
| **Congenital cardiac abnormalities**  (based on information provided for 29/30 cohort members) | 9/29 (31%)  Description:   1. Coarctation of the aorta (repaired with end to end anastomosis at age 6m) 2. Congenital aortic valve stenosis (secondary to dysplastic valves with partial fusion of lateral right and non-coronary cusps) 3. Bicuspid aortic valve 4. Mild pulmonary valve stenosis 5. Small ventricular septal defect 6. Two ventricular septal defects with additional right ventricular hypertrophy (both completely resolved by 9 m) 7. Atrial septal defect 8. “Hole in the heart” (? patent ductus arteriosus or ASD) which closed spontaneously during first few days of life**.** 9. Mild peripheral pulmonary stenosis (gradient < 20mm Hg) |
| **Early-onset cardiomyopathy**  (based on information provided for 28/28 cohort members who survived > 3 months of age) | Early-onset dilated cardiomyopathy (DCM) developed in 2/28 (7%) cohort members. Age at diagnosis: 18m, 9y  Additional non-congenital cardiac abnormalities developed in 3/28 (11%) additional cohort members:   1. Mild left ventricular dysfunction, mild mitral valve prolapse with regurgitation and possible hidden right to left shunt diagnosed in childhood 2. Mild hypocontractility of left ventricle diagnosed at age 3y (ejection fraction 50%), 3. Dilated right ventricle at autopsy following death at age 13 from pneumonia (may have been secondary to respiratory failure)   Overall number of cohort members with non-congenital cardiac abnormalities = 5/28 (18%) |
| **Cardiac abnormalities - total** | Overall number of cohort members with congenital (total 9 cases) and/or early-onset cardiac abnormalities (total 5 cases) = 13/28 (46%)  Note: 1 case with both congenital and non-congenital cardiac abnormalities (bicuspid aortic valve, and DCM: onset 18m) |
| **Foot features (age at onset)** |  |
| **Pes cavus**  (based on information provided for 22/28 cohort members who survived > 3 months) | 1/22 (5%) |
| **Pes planus**  (based on information provided for 21/28 cohort members who survived > 3 months) | 5/21 (24%) |
| **Other foot abnormalities** | At least 9/21 (43%)  Description:   1. Equinovarus foot deformities (residual congenital talipes in some cases) = at least 4 (%) 2. Calcaneovalgus foot deformities (residual congenital talipes in some cases) = at least 2(%)   Note: Need for “in shoe” orthotics / calipers / ankle foot orthoses = at least 3 (%) |
| **Bone features** |  |
| **Osteopaenia (age at onset)**  (based on information provided for 13/27 cohort members who survived > 9 months) | 4/13 (31%)  Note: 1 case = 1^st^ noted age 4 years in a cohort member who also had growth hormone deficiency  Note: Many cohort members had not been screened for osteopaenia |
| **Pathological fractures (age at onset)**  (based on information provided for 18/27 cohort members who survived > 9 months) | 2/18 (11%) |
| **History of delayed bone age?**  (based on information provided for 11/27 cohort members who survived > 9 months) | 1/11 (9%)  Note: most cohort members had had not been screened (as no clinical indication) |
| **Additional features** |  |
| **Significant fatigability**  (based on information provided for 16/27 cohort members who survived > 3 months and not reliant on full time ventilation) | 11/16 (69%)  Mild to severe  Most debilitating symptom in at least 1 case. Resulted in increased evening falls in 1 case. |
| **Height and/or weight at or below 3^rd^ percentile**  (based on information provided for 19/29 cohort members who survived > day 1) | 5/19 (26%)  Descriptions:   1. Short stature since early childhood 2. Weight consistently less than 3^rd^ percentile 3. Always below the 3^rd^ percentile for both height and weight 4. Failure to thrive in infancy: short stature with height less than 3^rd^ percentile. Weight on 3^rd^ percentile until 2y3m then fell below 3^rd^ percentile. 5. Birth weight and head circumference 3^rd^ percentile, with birth length 50-90^th^ percentile in infant who dies at 3 months |
| **Joint hypermobility/ligamentous laxity**  (based on information provided for 22/29 cohort members who survived > day 1) | 16/22 (73%)  Descriptions:   1. Multiple limb joints / generalised (proximal and distal) = 5/22 (23%) (at least one more pronounced proximally) 2. Fingers, wrists, and/or feet only (distal): 8/22 (36%) 3. Elbows only = 2/22 (9%) 4. Distribution description not available = 1/22 (5%) |
| **Intellectual disability**  (based on information provided for 27/27 cohort members who survived > 9 months) | 2/27 (7%)  Note: mild intellectual disability reported in just two siblings from same family: one with additional diagnosis of attention deficit hyperactivity disorder |
| **NMD-or cardiac-related surgeries / non-imaging-related interventions that required a general anaesthetic**  (based on information provided for 26/29 cohort members who survived > day 1) | At least 10/26 (38%)  One cohort member had undergone four separate surgical procedures, and several had undergone two or more procedures.  Descriptions of surgical procedures and other interventions required:   1. Coarctation of aorta repair 2. Cleft palate repair 3. Tendon release surgery 4. Talipes correction surgery 5. Pterygium release surgery 6. Hip osteotomy 7. Spinal surgery/fusion 8. Insertion of tracheostomy (at least 3) 9. Insertion of parenteral gastrostomy tube 10. Bronchoscopies |
| **Creatine kinase (CK) results** |  |
| **CK**  (based on information provided for 26/29 cohort members who survived > day 1) | Normal = 23/26 (88%)  Elevated = 3/26 (12%)  (elevated readings: 2324 U/L, 500-1400 U/L, “twice upper limit of normal”) |
| **Electrophysiology results** |  |
| **Nerve conduction studies (NCS)**  (based on information provided for 18/29 cohort members who survived > day 1) | Normal = 14/18 (78%)  Essentially normal = 2/18 (11%)  Normal conduction but reduced CMAP amplitudes = 2/18 (11%)  Note: no decrement on repetitive nerve stimulation reported in subset of cohort members tested |
| **Electromyogram**  (based on information provided for 20/29 cohort members who survived > day 1) | Normal = 9/20 (45%)  Essentially normal = 1/20 (5%)  Equivocally abnormal: not otherwise specified = 1/20 (5%)  Myopathic = 8/20 (40%)  Neuropathic (in upper limbs only: lower limbs normal) = 1/20 (5%) (in a severely affected who died at 3 months of age) |
| **MRI Lower Limbs** |  |
| (based on information provided for 3/28 cohort members who survived > 3 months) | Data available on 3 cohort members (undertaken ages 3y, 9y and 15 years). Original imaging available from one of the three cases. Summary of findings provided in results section |
| **MRI Brain** |  |
| (based on information provided for 12/29 cohort members who survived > day 1) | Normal MRI Brain = 7/12 (58%)  Abnormal MRI Brain = 5/12 (42%)  3 cohort members with minor structural abnormalities:   1. **At age 22m**: Prominent CSF spaces with subjectively diminished white matter volume. Mild cerebellar ectopia and right plagiocephaly. Together with the superior position of the posterior arch of C1, this results in narrowing of the CSF spaces at the craniocervical junction. 2. **At 1y**: mild hypoplasia of the posterior corpus callosum. Result otherwise unremarkable.   **At 3y in same child:** Again, relatively small volume of the splenium compared to the genu with an incomplete appearing shape, compatible with mild dysplasia. Stable unusual shape of splenium of corpus callosum, may be within the range of normal variation, but cannot exclude mild dysplasia.   1. **At 16y (in a child born at 37 weeks gestation):** Periventricular hyperintensities   2 cohort members with birth hypoxia/trauma-related abnormalities:   1. **At 7d in a profoundly hypotonic infant following a complicated resuscitation at birth**: Mild signal abnormalities in posterior basal ganglia and lateral thalami suggestive of hypoxic-ischaemic injury. Normal brain morphology. 2. **In neonatal period in a profoundly hypotonic infant following resuscitation at birth**: Grade 2 intraventricular haemorrhage but normal brain morphology   Note: None of the cohort members with abnormal MRI brain results were born prematurely. |
| **MRI spine** |  |
| (based on information provided for 5/29 cohort members) | Normal in 5/5 cases  (Note: all focussed on vertebral column and spinal cord: none targeted to parapsinal muscles) |

**Legend**

m = months, y = years, d = day

“At least” indicates that ascertainment for item was incomplete, as item was not specifically requested during data ascertainment

CMAP = compound muscle action potential

**Reference cited**

[22] Ceyhan-Birsoy, O., et al., *Recessive truncating titin gene, TTN, mutations presenting as centronuclear myopathy.* Neurology, 2013. **81**(14): p. 1205-14
