# Supplementary material for: Congenital Titinopathy: Comprehensive characterization and pathogenic insights
Source: Ann Neurol. 2018 Jul 27;83(6):1105–24. doi: 10.1002/ana.25241 (PMC6105519; doi:10.1002/ana.25241)
Supplement: Supplementary file 4 — Supporting Information [file ANA-83-1105-s004.docx]

**Supplementary Table 3: Comparison of segregation-inconclusive cases (blue: Families 28-31) and patients with one “metatranscript-only” mutation (yellow: Families 21-27) with features seen in clinical cohort**

Note1: “Metatranscript-only” patients were included in the clinical cohort; segregation-inconclusive cases were not included in the clinical cohort

Note 2: Additional clinical feature information, including additional percentages, is provided In Tables 2 and 3 and Supplementary Table 2

Abbreviations: F = Family e.g. F25 = Family 25, S1 = oldest of two affected siblings, S2 = youngest of two affected siblings, UL = upper limb, LL = lower limb, UL & LL = both upper and lower limb involvement: neither predominant, P = proximal, D = distal, P & D = proximal and distal muscle involvement: neither predominant, PDA = patent ductus arteriosus, ASD = atrial septal defect

For comparison columns: Y = Yes feature present, N = No feature not present, U = Unknown if feature present, ND = investigation not done, N/A = not assessable

|  |  | | | | | | | | | | | | | |
| --- | --- | --- | --- | --- | --- | --- | --- | --- | --- | --- | --- | --- | --- | --- |
| **Features** | **F28** | **F29** | **F30** | **F31** | **F20**  **S1** | **F20 S2** | **F21** | **F22** | **F23** | **F24** | **F25** | **F26**  **S1** | **F26 S2** | **F27** |
| **Age** | **18y** | **Early 40s** | **8y** | **33y** | **14y** | **2y6m** | **2y5m** | **7y** | **4y3m** | **6y** | **8y** | **18y** | **13y** | **14y** |
| **Common clinical features**  **(present in 50% or more of cohort members)** |  |  |  |  |  |  |  |  |  |  |  |  |  |  |
| **Common pregnancy features** |  |  |  |  |  |  |  |  |  |  |  |  |  |  |
| **Reduced foetal movements** | **U** | **N** | **N** | **Y** | **Y** | **Y** | **Y** | **N** | **Y** | **Y** | **Y** | **Y** | **Y** | **N** |
| **Common congenital features** |  |  |  |  |  |  |  |  |  |  |  |  |  |  |
| **Congenital hypotonia/weakness** | **U** | **Y** | **Y** | **Y** | **Y** | **Y** | **Y** | **Y** | **Y** | **Y** | **Y** | **Y** | **Y** | **Y** |
| **One or more congenital limb contractures** | **U** | **N** | **N** | **Y** | **Y** | **Y** | **Y** | **N** | **Y** | **Y** | **Y** | **Y** | **Y** | **Y** |
| **Neonatal feeding difficulties** | **U** | **N** | **U** | **N** | **Y** | **N** | **Y** | **Y** | **Y** | **N** | **Y** | **Y** | **Y** | **N** |
| **Common motor features** |  |  |  |  |  |  |  |  |  |  |  |  |  |  |
| **Delayed sitting** | **U** | **Y** | **U** | **U** | **Y** | **N/A** | **Y** | **N** | **N** | **N** | **N/A** | **Y** | **U** | **N** |
| **Currently or previously able to walk independently** | **Y** | **Y** | **Y** | **Y** | **Y** | **N** | **Y** | **Y** | **Y** | **Y** | **N** | **Y** | **Y** | **Y** |
| **If walking: positive Gowers’ manoeuvre** | **Y** | **U** | **N** | **Y** | **Y** | **N/A** | **Y** | **Y** | **N** | **Y** | **N/A** | **Y** | **Y** | **Y** |
| **Common muscle bulk features** |  |  |  |  |  |  |  |  |  |  |  |  |  |  |
| **Generalised muscle hypotrophy/wasting** | **Y** | **Y** | **U** | **N** | **Y** | **U** | **N** | **Y** | **N** | **Y** | **U** | **U** | **U** | **Y** |
| **Absence of muscle hypertrophy pseudohypertrophy** | **Y** | **Y** | **Y** | **U** | **Y** | **Y** | **Y** | **Y** | **Y** | **Y** | **U** | **U** | **U** | **Y** |
| **Common limb features** |  |  |  |  |  |  |  |  |  |  |  |  |  |  |
| **Symmetrical limb weakness** | **Y** | **Y** | **Y** | **Y** | **N** | **N** | **Y** | **Y** | **Y** | **Y** | **Y** | **U** | **U** | **Y** |
| **Mild to moderate limb weakness (MRC 3-4/5)** | **N** | **Y** | **N** | **Y** | **Y** | **N** | **Y** | **Y** | **Y** | **N** | **N** | **Y** | **Y** | **Y** |
| **UL & LL weakness approximately equal** | **N** | **Y** | **Y** | **N** | **N** | **N** | **Y** | **Y** | **N** | **Y** | **Y** | **U** | **U** | **Y** |
| **One or more acquired limb contractures** | **Y** | **Y** | **Y** | **Y** | **Y** | **N** | **Y** | **Y** | **Y** | **N** | **U** | **Y** | **N** | **Y** |
| **Reduced, trace or absent reflexes** | **Y** | **Y** | **U** | **U** | **Y** | **Y** | **Y** | **Y** | **Y** | **Y** | **U** | **U** | **U** | **Y** |
| **Common facial features** |  |  |  |  |  |  |  |  |  |  |  |  |  |  |
| **Absence of ophthalmoplegia** | **Y** | **Y** | **Y** | **Y** | **Y** | **Y** | **Y** | **Y** | **Y** | **Y** | **U** | **Y** | **Y** | **Y** |
| **Facial weakness** | **Y** | **Y** | **Y** | **Y** | **Y** | **Y** | **Y** | **Y** | **Y** | **Y** | **U** | **Y** | **N** | **N** |
| **High arched palate** | **Y** | **N** | **U** | **U** | **Y** | **N** | **Y** | **Y** | **Y** | **N** | **U** | **Y** | **Y** | **N** |
| **Common neck & spinal (axial) features** |  |  |  |  |  |  |  |  |  |  |  |  |  |  |
| **Neck flexion weakness** | **Y** | **Y** | **U** | **Y** | **Y** | **Y** | **U** | **Y** | **Y** | **Y** | **Y** | **U** | **U** | **Y** |
| **Neck extension weakness** | **N** | **N** | **U** | **N** | **Y** | **Y** | **Y** | **U** | **U** | **N** | **Y** | **Y** | **U** | **N** |
| **Scoliosis** | **Y** | **N** | **N** | **N** | **Y** | **N** | **N** | **Y** | **Y** | **N** | **N** | **Y** | **Y** | **N** |
| **Common respiratory features** |  |  |  |  |  |  |  |  |  |  |  |  |  |  |
| **Objective evidence of respiratory insufficiency**  **(1)** FVC 77% at 7y **(2)** FVC 900ml at 18y | **Y** | **U** | **Y** | **N** | **N** | **N** | **N** | **Y (1)** | **N** | **N** | **N** | **Y (2)** | **N** | **N** |
| **Objective evidence of respiratory insufficiency & scoliosis, chest wall deformity or both** | **Y** | **U** | **N** | **N** | **N** | **N** | **N** | **Y** | **N** | **N** | **N** | **Y** | **N** | **N** |
| **Common additional features** |  |  |  |  |  |  |  |  |  |  |  |  |  |  |
| **Joint hypermobility** | **Y** | **N** | **N** | **N** | **Y** | **Y** | **Y** | **N** | **Y** | **Y** | **Y** | **Y** | **Y** | **Y** |
| **Absence of cognitive involvement** | **Y** | **Y** | **Y** | **Y** | **Y** | **Y** | **Y** | **Y** | **Y** | **Y** | **Y** | **N** | **N** | **Y** |
| **Common investigation results** |  |  |  |  |  |  |  |  |  |  |  |  |  |  |
| **Normal creatine kinase (CK) level** | **N** | **Y** | **Y** | **Y** | **Y** | **Y** | **Y** | **Y** | **Y** | **Y** | **Y** | **Y** | **U** | **Y** |
| **Normal electromyogram result** | **Y** | **U** | **N** | **Y** | **N** | **ND** | **N** | **N** | **Y** | **N** | **N** | **Y** | **ND** | **U** |
| **Normal nerve conduction study (NCS) result** | **Y** | **U** | **U** | **Y** | **N** | **ND** | **Y** | **Y** | **Y** | **Y** | **Y** | **Y** | **ND** | **Y** |
| **Extended clinical features**  **(present in two or more, but less than 50% of cohort members)** | | | | | | | | | | | | | | |
| **Family history of cardiomyopathy** | **N** | **Y** | **N** | **N** | **N** | **N** | **N** | **N** | **N** | **N** | **N** | **N** | **N** | **N** |
| **Pregnancy features** |  |  |  |  |  |  |  |  |  |  |  |  |  |  |
| **Abnormal liquor volume** | **U** | **N** | **U** | **Y** | **U** | **U** | **Y** | **Y** | **Y** | **N** | **U** | **Y** | **Y** | **N** |
| **Intrauterine growth retardation** | **U** | **N** | **N** | **N** | **N** | **N** | **N** | **N** | **N** | **N** | **N** | **Y** | **Y** | **N** |
| **Prenatally detected contractures** | **U** | **N** | **N** | **N** | **N** | **N** | **N** | **N** | **N** | **N** | **Y** | **N** | **N** | **N** |
| **Preterm delivery** | **N** | **N** | **Y** | **N** | **Y** | **N** | **N** | **N** | **N** | **N** | **N** | **N** | **Y** | **N** |
| **Breech or other abnormal presentation** | **N** | **N** | **N** | **Y** | **N** | **N** | **Y** | **N** | **N** | **Y** | **N** | **Y** | **U** | **N** |
| **Congenital & infant features** |  |  |  |  |  |  |  |  |  |  |  |  |  |  |
| **Features first noted at birth** | **Y** | **Y** | **Y** | **N** | **N** | **N** | **N** | **Y** | **N** | **N** | **N** | **N** | **N** | **Y** |
| **Features first noted in infancy** | **N** | **N** | **N** | **N** | **N** | **N** | **N** | **N** | **N** | **N** | **N** | **N** | **N** | **N** |
| **Congenital limb contractures involving two or more areas (“Arthrogryposis multiplex congenita”)** | **N** | **N** | **N** | **N** | **Y** | **Y** | **Y** | **N** | **Y** | **Y** | **Y** | **Y** | **Y** | **N** |
| **Congenital finger contracture(s)** | **U** | **N** | **N** | **N** | **N** | **N** | **Y** | **N** | **Y** | **N** | **Y** | **Y** | **U** | **N** |
| **Congenital wrist contracture(s)** | **U** | **N** | **N** | **N** | **N** | **Y** | **Y** | **N** | **Y** | **Y** | **N** | **Y** | **U** | **N** |
| **Congenital elbow contracture(s)** | **U** | **N** | **N** | **N** | **Y** | **N** | **Y** | **N** | **N** | **N** | **Y** | **N** | **Y** | **Y** |
| **Congenital hip contracture(s)** | **U** | **N** | **N** | **N** | **N** | **N** | **Y** | **N** | **N** | **N** | **N** | **N** | **N** | **N** |
| **Congenital dysplasia of hips (CDH)** | **U** | **N** | **N** | **N** | **N** | **N** | **N** | **N** | **Y** | **N** | **N** | **N** | **N** | **N** |
| **Congenital knee contracture(s)** | **U** | **N** | **N** | **N** | **N** | **Y** | **Y** | **N** | **N** | **N** | **N** | **N** | **N** | **N** |
| **Congenital ankle contracture(s)/talipes** | **U** | **N** | **N** | **Y** | **Y** | **N** | **Y** | **N** | **Y** | **Y** | **Y** | **N** | **N** | **N** |
| **Congenital scoliosis** | **U** | **N** | **N** | **N** | **N** | **N** | **N** | **N** | **N** | **N** | **U** | **N** | **N** | **N** |
| **Congenital fractures** | **N** | **N** | **N** | **N** | **N** | **N** | **N** | **N** | **N** | **N** | **U** | **Y** | **N** | **N** |
| **Congenital cardiac abnormalities** | **N** | **N** | **Y** | **N** | **N** | **N** | **N** | **N** | **N** | **N** | **Y** | **N** | **N** | **N** |
| **Neonatal respiratory difficulties** | **N** | **N** | **U** | **N** | **N** | **Y** | **N** | **N** | **N** | **N** | **Y** | **Y** | **Y** | **N** |
| **One or > additional congenital anomalies (facial/syndromal)** | **U** | **N** | **U** | **N** | **N** | **N** | **Y** | **Y** | **N** | **N** | **Y** | **Y** | **Y** | **U** |
| **Facial & syndromal features** |  |  |  |  |  |  |  |  |  |  |  |  |  |  |
| **Ptosis** | **N** | **N** | **N** | **N** | **N** | **N** | **N** | **Y** | **Y** | **N** | **U** | **N** | **Y** | **N** |
| **Elongated face** | **N** | **N** | **N** | **N** | **N** | **N** | **N** | **N** | **N** | **N** | **N** | **N** | **Y** | **N** |
| **Asymmetrical facial tissue** | **Y** | **N** | **N** | **N** | **N** | **N** | **Y** | **N** | **N** | **N** | **N** | **N** | **N** | **N** |
| **Palatal Cleft** | **N** | **N** | **N** | **N** | **N** | **N** | **N** | **Y** | **N** | **N** | **N** | **N** | **N** | **N** |
| **Short palpebral fissures** | **N** | **N** | **N** | **N** | **N** | **N** | **N** | **N** | **N** | **N** | **N** | **Y** | **N** | **N** |
| **Short/small mouth** | **N** | **N** | **N** | **N** | **Y** | **N** | **N** | **N** | **N** | **N** | **N** | **N** | **N** | **N** |
| **Retrognathia or micrognathia** | **N** | **N** | **N** | **N** | **N** | **N** | **N** | **N** | **N** | **N** | **N** | **N** | **N** | **N** |
| **Torticollis** | **N** | **N** | **N** | **N** | **N** | **N** | **Y** | **N** | **N** | **N** | **N** | **N** | **N** | **N** |
| **Plagiocephaly** | **N** | **N** | **N** | **N** | **N** | **N** | **N** | **N** | **N** | **N** | **N** | **N** | **N** | **N** |
| **Reduced or abnormal palmar creases** | **N** | **U** | **U** | **N** | **N** | **N** | **Y** | **N** | **N** | **U** | **Y** | **N** | **Y** | **N** |
| **Undescended testes** | **U** | **U** | **U** | **U** | **N** | **N** | **N** | **N** | **N** | **N** | **N** | **Y** | **Y** | **N** |
| **Height and weight below 3^rd^ percentile** | **Y** | **U** | **U** | **N** | **Y** | **N** | **N** | **N** | **N** | **N** | **N** | **N** | **N** | **U** |
| **Pattern & severity of limb involvement** |  |  |  |  |  |  |  |  |  |  |  |  |  |  |
| **LL predominant weakness** | **Y** | **N** | **N** | **N** | **N** | **N** | **N** | **N** | **Y** | **N** | **N** | **U** | **U** | **N** |
| **UL predominant weakness** | **N** | **N** | **N** | **Y** | **Y** | **Y** | **N** | **N** | **N** | **N** | **N** | **U** | **U** | **N** |
| **P predominant weakness** | **N** | **N** | **Y** | **Y** | **N** | **U** | **Y** | **N** | **Y** | **Y** | **Y** | **Y** | **Y** | **N** |
| **P & D muscles equally involved** | **N** | **Y** | **N** | **N** | **N** | **U** | **N** | **Y** | **N** | **N** | **N** | **N** | **N** | **N** |
| **D predominant weakness** | **Y** | **N** | **N** | **N** | **Y** | **U** | **N** | **N** | **N** | **N** | **N** | **N** | **N** | **N** |
| **Mild weakness (MRC grade 4/5 or more)** | **Y** | **N** | **Y** | **N** | **N** | **Y** | **N** | **N** | **N** | **Y** | **N** | **N** | **N** | **N** |
| **Moderate weakness (MRC grade 3/5)** | **N** | **N** | **N** | **N** | **N** | **N** | **N** | **N** | **N** | **N** | **Y** | **N** | **N** | **N** |
| **Mild to severe weakness (MRC 2-4/5)** | **N** | **N** | **N** | **N** | **N** | **N** | **N** | **N** | **N** | **N** | **N** | **N** | **N** | **N** |
| **Non-congenital limb contractures** |  |  |  |  |  |  |  |  |  |  |  |  |  |  |
| **2 or > non-congenital limb contractures** | **N** | **Y** | **N** | **Y** | **Y** | **N** | **N** | **Y** | **Y** | **N** | **U** | **U** | **U** | **Y** |
| **Non-congenital ankle contracture(s)** | **Y** | **Y** | **Y** | **Y** | **Y** | **N** | **N** | **Y** | **Y** | **N** | **U** | **U** | **U** | **Y** |
| **Non-congenital knee contracture(s)** | **N** | **Y** | **N** | **Y** | **N** | **N** | **N** | **Y** | **N** | **N** | **U** | **U** | **U** | **N** |
| **Non-congenital hip contracture(s)** | **N** | **Y** | **N** | **N** | **N** | **N** | **N** | **N** | **N** | **N** | **U** | **U** | **U** | **N** |
| **Non-congenital shoulder contracture(s)** | **N** | **U** | **N** | **N** | **N** | **N** | **N** | **N** | **N** | **N** | **U** | **U** | **U** | **N** |
| **Non-congenital elbow contracture(s)** | **N** | **N** | **N** | **Y** | **Y** | **N** | **N** | **Y** | **N** | **N** | **U** | **Y** | **N** | **Y** |
| **Non-congenital wrist/hand/finger contracture(s)** | **N** | **N** | **N** | **Y** | **N** | **N** | **Y** | **Y** | **Y** | **N** | **U** | **N** | **U** | **N** |
| **Neck, spinal, chest wall & scapular features** |  |  |  |  |  |  |  |  |  |  |  |  |  |  |
| **Limited range of neck movement** | **Y** | **U** | **U** | **U** | **N** | **N** | **U** | **N** | **N** | **N** | **N** | **U** | **U** | **N** |
| **Kyphosis** | **Y** | **U** | **N** | **Y** | **N** | **N** | **N** | **N** | **N** | **N** | **N** | **U** | **U** | **N** |
| **Spinal rigidity** | **Y** | **U** | **U** | **Y** | **N** | **N** | **N** | **Y** | **Y** | **N** | **N** | **Y** | **Y** | **N** |
| **Lumbar hyperlordosis** | **Y** | **U** | **U** | **Y** | **Y** | **N** | **N** | **N** | **N** | **Y** | **N** | **U** | **U** | **N** |
| **Chest wall deformity** | **Y** | **N** | **N** | **Y** | **N** | **N** | **Y** | **N** | **N** | **N** | **Y** | **U** | **U** | **N** |
| **Scapular winging** | **N** | **N** | **U** | **Y** | **Y** | **N** | **Y** | **N** | **N** | **Y** | **Y** | **Y** | **Y** | **Y** |
| **Respiratory features** |  |  |  |  |  |  |  |  |  |  |  |  |  |  |
| **Nocturnal ventilation requirement** | **N*** | **N** | **Y** | **N** | **N** | **N** | **N** | **N** | **N** | **N** | **N** | **N** | **N** | **N** |
| **Frequent and/or severe respiratory infections** | **N** | **U** | **N** | **N** | **N** | **N** | **N** | **N** | **Y** | **N** | **N** | **N** | **N** | **N** |
| **Weak cough** | **N** | **U** | **U** | **N** | **N** | **N** | **N** | **N** | **N** | **N** | **Y** | **U** | **U** | **N** |
| **Paradoxical breathing pattern?** | **N** | **U** | **U** | **N** | **N** | **N** | **U** | **N** | **N** | **N** | **U** | **N** | **N** | **N** |
| **Cardiac features** |  |  |  |  |  |  |  |  |  |  |  |  |  |  |
| **Congenital cardiac abnormalities** | **N** | **N** | **Y** | **N** | **N** | **N** | **N** | **N** | **N** | **N** | **Y** | **N** | **N** | **N** |
| **Non-congenital cardiac abnormalities** | **N*** | **Y** | **Y** | **N** | **N** | **N** | **N** | **N** | **N** | **N** | **N** | **N** | **N** | **N** |
| **Non-congenital dilated cardiomyopathy** | **N** | **Y** | **N** | **N** | **N** | **N** | **N** | **N** | **N** | **N** | **N** | **N** | **N** | **N** |
| **Left ventricular dysfunction/hyocontractility** | **N** | **N** | **Y** | **N** | **N** | **N** | **N** | **N** | **N** | **N** | **N** | **N** | **N** | **N** |
| **Congenital &/or non-congenital cardiac anomalies** | **N** | **Y** | **Y** | **N** | **N** | **N** | **N** | **N** | **N** | **N** | **Y** | **N** | **N** | **N** |
| **Foot features** |  |  |  |  |  |  |  |  |  |  |  |  |  |  |
| **Pes planus** | **N** | **N** | **U** | **N** | **N** | **N** | **N** | **N** | **N** | **N** | **Y** | **U** | **U** | **N** |
| **Other foot deformities** | **Y** | **N** | **U** | **N** | **Y** | **N** | **N** | **N** | **N** | **Y** | **Y** | **U** | **U** | **N** |
| **Motor development & ambulation** |  |  |  |  |  |  |  |  |  |  |  |  |  |  |
| **Delayed walking** | **Y** | **Y** | **Y** | **U** | **Y** | **U** | **Y** | **Y** | **N** | **Y** | **N** | **Y** | **Y** | **N** |
| **Not able to and unlikely to ever walk** | **N** | **N** | **N** | **N** | **N** | **N** | **N** | **N** | **N** | **N** | **Y** | **N** | **N** | **N** |
| **Age <5y, not walking but may yet do so** | **N** | **N** | **N** | **N** | **N** | **Y** | **N** | **N** | **N** | **N** | **N** | **N** | **N** | **N** |
| **If walking: Ever able to walk fast or run?** | **Y** | **U** | **Y** | **Y** | **N** | **N/A** | **Y** | **N** | **Y** | **Y** | **N/A** | **N** | **N** | **Y** |
| **If walking: Ever able to jump?** | **Y** | **U** | **U** | **U** | **N** | **N/A** | **N** | **N** | **N** | **Y** | **N/A** | **N** | **N** | **U** |
| **Stable or slow loss of limb weakness/walking ability** | **Y** | **Y** | **N** | **Y** | **Y** | **N** | **N** | **Y** | **Y** | **Y** | **N/A** | **Y** | **N** | **Y** |
| **Currently having marked walking difficulties** | **Y** | **N** | **N** | **N** | **N** | **N** | **N** | **N** | **N** | **N** | **N/A** | **N** | **N** | **N** |
| **For cohort members who are/were able to walk:**  **Rate of loss of ambulation** |  |  |  |  |  |  |  |  |  |  |  |  |  |  |
| **Moderate rate of loss of ambulation** | **N** | **N** | **N** | **N** | **N** | **N** | **N** | **N** | **N** | **N** | **N/A** | **N** | **N** | **N** |
| **Walking & still gaining ambulatory ability** | **N** | **N** | **Y** | **N** | **N** | **Y** | **Y** | **N** | **N** | **N** | **N/A** | **N** | **Y** | **N** |
| **Feeding abnormalities & voice** |  |  |  |  |  |  |  |  |  |  |  |  |  |  |
| **Ongoing chewing and swallowing difficulties** | **Y** | **U** | **N** | **N** | **Y** | **N** | **N** | **N** | **N** | **N** | **N** | **Y** | **U** | **N** |
| **Current need for NGT or PEG feeding** | **N** | **N** | **U** | **U** | **N** | **N** | **N** | **N** | **Y** | **N** | **N** | **Y** | **U** | **N** |
| **Nasal voice** | **N** | **N** | **U** | **U** | **Y** | **N** | **N** | **N** | **N** | **N** | **N** | **U** | **U** | **N** |
| **Fatigability** |  |  |  |  |  |  |  |  |  |  |  |  |  |  |
| **Mild, moderate to severe fatigability** | **U** | **U** | **N** | **N** | **Y** | **N** | **N** | **Y** | **N** | **Y** | **U** | **U** | **Y** | **U** |
| **Bone health** |  |  |  |  |  |  |  |  |  |  |  |  |  |  |
| **Osteopaenia** | **U** | **U** | **U** | **U** | **U** | **U** | **N** | **N** | **N** | **N** | **U** | **N** | **N** | **U** |
| **Pathological fractures** | **N** | **N** | **N** | **N** | **N** | **N** | **N** | **N** | **N** | **N** | **U** | **N** | **N** | **N** |
| **Investigation results** |  |  |  |  |  |  |  |  |  |  |  |  |  |  |
| **Myopathic EMG** | **N** | **U** | **Y** | **N** | **Y** | **ND** | **Y** | **Y** | **N** | **Y** | **Y** | **N** | **ND** | **U** |
| **Normal MRI Brain** | **ND** | **ND** | **ND** | **ND** | **Y** | **ND** | **Y** | **ND** | **N** | **ND** | **Y** | **N** | **ND** | **ND** |
| **Abnormal MRI brain (variable findings)** | **ND** | **ND** | **ND** | **ND** | **N** | **ND** | **N** | **ND** | **Y** | **ND** | **N** | **Y** | **ND** | **ND** |
| **Normal spinal cord and vertebral bodies on MRI imaging** | **Y** | **ND** | **ND** | **ND** | **ND** | **ND** | **ND** | **ND** | **ND** | **ND** | **N** | **Y** | **ND** | **ND** |
| **Clear pattern of muscle involvement/ sparing seen on lower limb MRI** | **ND** | **ND** | **N** | **Y** | **ND** | **ND** | **ND** | **ND** | **Y** | **ND** | **ND** | **Y** | **ND** | **Y** |
